# Isorhamnetin Regulates Programmed Death Ligand-1 Expression by Suppressing the EGFR–STAT3 Signaling Pathway in Canine Mammary Tumors

**DOI:** 10.3390/ijms25010670

**Published:** 2024-01-04

**Authors:** Chen Mei, Xue Zhang, Yan Zhi, Zhixuan Liang, Haojun Xu, Zhenyi Liu, Ying Liu, Yanli Lyu, Hongjun Wang

**Affiliations:** 1 Institute of Animal Husbandry and Veterinary Medicine, Beijing Academy of Agriculture and Forestry Sciences, No. 11 Shuguanghuayuan Middle Road, Haidian District, Beijing 100097, China; bjmeichen2022@163.com (C.M.); zhangxue_531@163.com (X.Z.); bjzhiyan@gmail.com (Y.Z.); liangzhixuan0422@163.com (Z.L.); 15931563201@163.com (H.X.); liuzylynn@163.com (Z.L.); liuying@baafs.net.cn (Y.L.); 2 Department of Clinical Veterinary Medicine, College of Veterinary Medicine, China Agricultural University, No. 2 Yuanmingyuan West Road, Haidian District, Beijing 100193, China

**Keywords:** canine mammary tumor, isorhamnetin, surface plasmon resonance (SPR), target protein, programmed death ligand-1 (PD-L1), EGFR-STAT3 signaling pathway

## Abstract

Programmed death ligand-1 (PD-L1) is highly expressed in a variety of cancer cells and suggests a poorer prognosis for patients. The natural compound isorhamnetin (ISO) shows promise in treating cancers and causing damage to canine mammary tumor (CMT) cells. We investigated the mechanism of ISO in reducing PD-L1 expression in CMT cells. Clustered, regularly interspaced short palindromic repeat-associated protein 9 (CRISPR/Cas9) was used to mediate *CD274* knockout in U27 cells. Then, monoclonal cells were screened and cultured. Nucleotide sequencing and expression of PD-L1 were detected. Additionally, we examined cell migration, invasion, and damage. Immunofluorescent staining of PD-L1 was examined in U27 cells. The signaling pathways were measured by Western blotting. Murine xenotransplantation models and murine immunocompetent allograft mammary tumor models were established to evaluate the effect of ISO therapy. Expression of Ki-67, caspase3, and PD-L1 were analyzed by immunohistochemistry. A pull-down assay was used to explore which proteins could bind to ISO. Canine EGFR protein was purified and used to detect whether it directly binds to ISO using a surface plasmon resonance assay. ISO inhibited the EGFR-STAT3-PD-L1 signaling pathway and blocked cancer growth, significantly increasing the survival rate of healthy cells. The cell membrane receptor EGFR was identified as a direct target of ISO. ISO could be exploited as an antineoplastic treatment of CMT by targeting EGFR to suppress PD-L1 expression.

## 1. Introduction

As companion animals, dogs inhabit the same environment as humans and are exposed to the same carcinogenic factors [1,2]. In fact, environmental toxins, old age, and obesity are common carcinogenic factors in both humans and dogs [3]. Therefore, canine spontaneous tumors are an ideal model for human tumor research [4]. Canine mammary tumors are the most common type of tumor in female dogs, accounting for ~25–42% of all canine tumors, of which ~50% are malignant [5]. As dogs age, canine mammary tumors become more prevalent, and traditional surgical and chemotherapeutic approaches often cause tumor cells to develop resistance, negatively impacting prognosis [6]. Therefore, there is an urgent need to find and develop new drugs to treat canine mammary tumors.

Epidermal growth factor receptor (EGFR) is a member of the epidermal growth factor receptor family and comprises an extracellular ligand-binding region, a transmembrane region, and an intracellular kinase region [7]. It is widely distributed on the surface of mammalian cells and has an important role in physiological processes, including the growth, proliferation, and differentiation of cancer cells [8]. Thus, alterations in the structure or expression of EGFR can lead to tumorigenesis [9]. The EGFR–STAT3–PD-L1 pathway has an important role in cancer growth and metastasis, and inhibiting its expression can effectively reduce cancer migration and invasion [10]. Research into PD-L1 is a hot topic in oncology, particularly for the treatment of breast cancer [11]. Inhibition of different types of lung cancer cells by preventing PD-L1 expression can be achieved by reducing the mitochondrial membrane potential to induce apoptosis in these cells [12]. In addition to lung cancer cells, PD-L1 also shows higher expression on the surface of some types of breast cancer cells, providing a possible potential treatment target.

**Figure 1:**
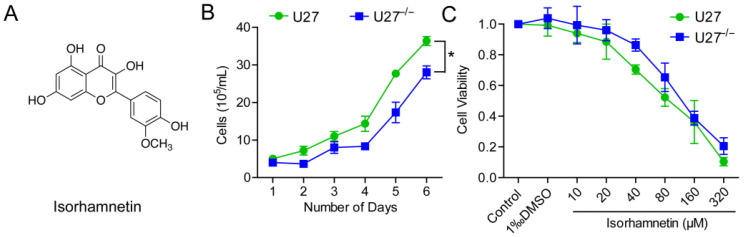
PD-L1 is involved in U27 cell growth. (**A**) Chemical structure of ISO. (**B**) U27 and U27^−/−^ cell growth curves. (**C**) Effect of different concentrations of ISO on viability of U27 and U27^−/−^ cells after 48 h. Data are mean ± SD. * *p* <0.05, using Student’s *t*-test.

There is increasing interest in the use of botanical compounds as anti-cancer agents because of their potential tumor cell selectivity and cytotoxicity, as well as fewer side effects [13]. Isorhamnetin (ISO)’s chemical formula is 3,5,7-trihydroxy-2-(4-hydroxy-3-methoxyphenyl)-4*H*-chromen-4-one (Figure 1A); it is a flavonoid extracted from fruits, vegetables, and tea leaves, and it is also widely found in plants such as Ginkgo biloba and Sichuan dome [14]. The molecular weight of ISO is 316.2623, and it is in the state of a light yellow needle-like crystal. It is used for cough, indigestion, abdominal pain, bruises and swelling, cardiovascular diseases, and hemorrhage. Also, its use for the prevention or treatment of colon cancers has been previously reported [15]. However, further investigations are required to gain a better understanding of the possible antitumor properties of ISO, given that its direct cellular targets and biological impacts remain largely unknown. In this study, we investigated the anti-tumor activity of ISO in canine mammary tumor cells, murine breast tumor cells, and nude mouse models. We identified EGFR as a direct target of ISO, suggesting that ISO could be utilized against EGFR-related pathologies, including cancer.

## 2. Results

### 2.1. PD-L1 Participates in U27 Cell Growth

To determine whether ISO causes cell damage by regulating *CD274* (also known as PD-L1, *B7-H1)*, we constructed a U27^−/−^ cell line with deletion of *CD274* by utilizing CRISPR/Cas9. Sanger sequencing and PCR were used to verify the successful knockout of *CD274* in CMT-U27 cell lines. The Western blot results showed that the expression level of PD-L1 protein in U27^−/−^ cells was 34.6% of that in U27 cells (Figure S1). Growth curves showed that U27^−/−^ cells grew at a slower rate compared with canine mammary tumor U27 cells and took longer to reach the logarithmic growth phase (Figure 1B).

The cell viability of U27 cells and U27^−/−^ cells was measured using a cell counting kit-8 assay (CCK8). After treatment for 48 h with different concentrations of ISO, the cell viability decreased to 50% with 128 μM ISO compared with the control group; thus, in subsequent experiments, 10, 20, and 40 μM ISO were used (Figure 1A,C).

### 2.2. Inhibition of PDL1 Expression in Cells by ISO Induces Cell Damage

**Figure 2:**
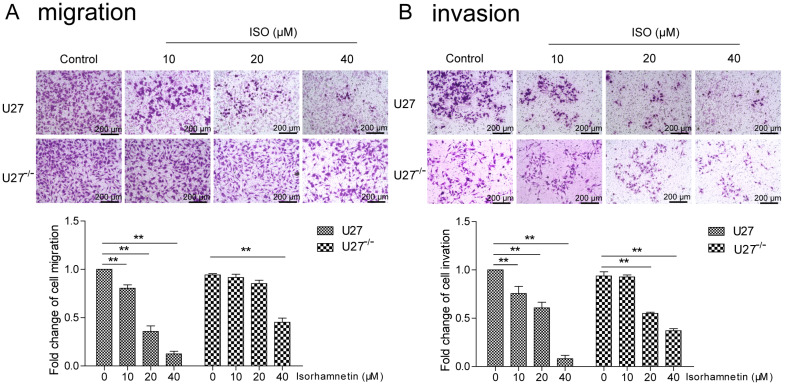
ISO inhibits U27 cell migration and invasion. Bar = 200 μm. (**A**) ISO inhibition of U27 cell migration increased with increasing ISO concentration, whereas inhibition of U27^−/−^ cell migration was only significant with 40 μM ISO. (**B**) Increasing concentrations of ISO inhibited U27 cell invasion but only had a significant effect on the invasive ability of U27^−/−^ cells at higher concentrations. Data are mean ± SD. ** *p* < 0.01 on one-way ANOVA.

We examined the effect of ISO on cell migration and invasion ability. ISO reduced U27 cell migration in a concentration-dependent manner (control group: 511 ± 11, 10 μM ISO group: 411 ± 35, 20 μM ISO group: 193 ± 41, 40 μM ISO group: 64 ± 25; Figure 2A), whereas U27^−/−^ cell migration was only significantly inhibited by higher concentrations of ISO (control group: 481 ± 8, 10 μM ISO group: 441 ± 31, 20 μM ISO group: 410 ± 36, 40 μM ISO group: 218 ± 41; Figure 2A). A similar pattern was seen for the effect of different concentrations of ISO on cell invasion (U27 cells: control group, 264 ± 15; 10 μM ISO group, 198 ± 26; 20 μM ISO group, 159 ± 20; 40 μM ISO group, 21 ± 14; U27^−/−^ cells: control group, 247 ± 8. 10 μM ISO group, 219 ± 17; 20 μM ISO group, 130 ± 18; 40 μM ISO group, 91 ± 12; Figure 2B).

Next, we examined the effect of ISO on the cellular mitochondrial membrane potential. Compared with the control group (Figure S3A), cell mitochondrial membrane integrity was disrupted, red fluorescence gradually decreased, and green fluorescence gradually increased with increasing ISO concentration (Figure S3B–D). The addition of gefitinib (Figure S3E) decreased the red fluorescence, which was consistent with the results of ISO treatment. Compared with the U27 group, at a 10 μM ISO concentration, the cells appeared similar to the control group (Figure S3G), suggesting that the sensitivity of cells to ISO drug was reduced after the knockdown of *CD274* and that ISO induced cell damage through *CD274*.

The terminal deoxynucleotidyl transferase-mediated dUTP nick end labeling (TUNEL) method was used to detect the effect of ISO on cellular DNA integrity. Compared with the control group, cellular DNA integrity was disrupted, and cellular red fluorescence gradually increased with increasing ISO concentration (Figure S4A). After the addition of gefitinib, red fluorescence increased, which was consistent with the outcomes of ISO treatment.

**Figure 3:**
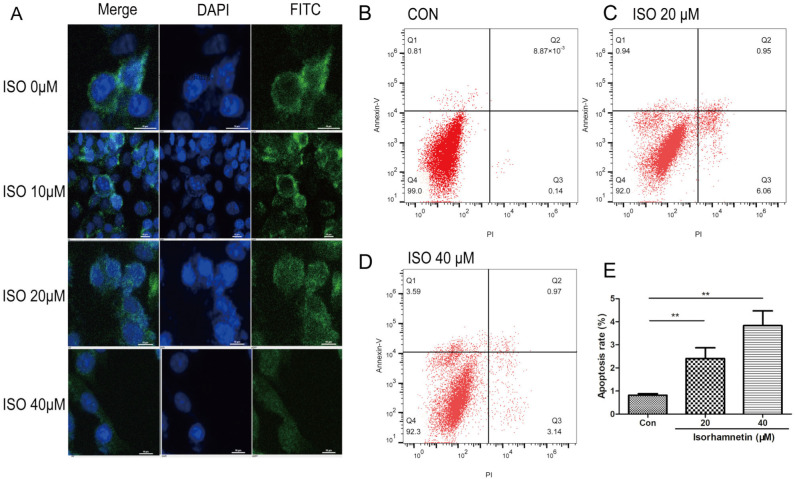
ISO decreased the expression of PD-L1 and induced cell apoptosis. (**A**) ISO inhibits PD-L1 expression on the membrane surface of U27 cells along a concentration gradient. With the increase in drug concentration, the expression of PD-L1 on the surface of cell membrane decreased (bar = 10 μm). (**B**–**E**) The effects of ISO on apoptosis were assessed using the Annexin V-FITC/PI assay in U27 cells treated with ISO for 48 h. The area of Q1 represented early apoptosis cells. The area of Q2 represented the late apoptosis cells. The area of Q3 represented the dead cells. The area of Q4 represented the living cells. (**B**) Control group. (**C**) U27 cell with 20 μM ISO. (**D**) U27 cell with 40 μM ISO. (**E**) Statistics of cell apoptosis. The data are representative results from three independent experiments and are expressed as the mean ± SD. ** *p* < 0.01.

Finally, cellular immunofluorescence was used to directly detect the effect of ISO on PD-L1 expression on the cell membrane surface. There was green fluorescence on the cell surface in the control group, indicating intact PD-L1 membrane protein expression (Figure 3). With the increase in ISO concentration, the green fluorescence gradually decreased, and the cells lost their basic morphology, becoming granular in shape.

The results of flow cytometry showed that the early and late apoptotic cell ratios were increased following ISO treatment. Compared to the control group, treatment with ISO led to an increase in the early apoptotic ratio (20 μM ISO, 0.93% ± 0.05% and 40 μM ISO, 0.95% ± 0.02%), late apoptotic ratio (20 μM ISO, 1.48% ± 0.49% and 40 μM ISO, 2.87% ± 0.64%), and total apoptotic ratio (20 μM ISO, 2.41% ± 0.46%; and 40 μM ISO, 3.84% ± 0.64%). Collectively, our findings suggested that ISO induced apoptosis in CMT-U27 cells.

### 2.3. EGFR-STAT3-PD-L1 Is Involved in the Induction of Cell Injury by ISO

**Figure 4:**
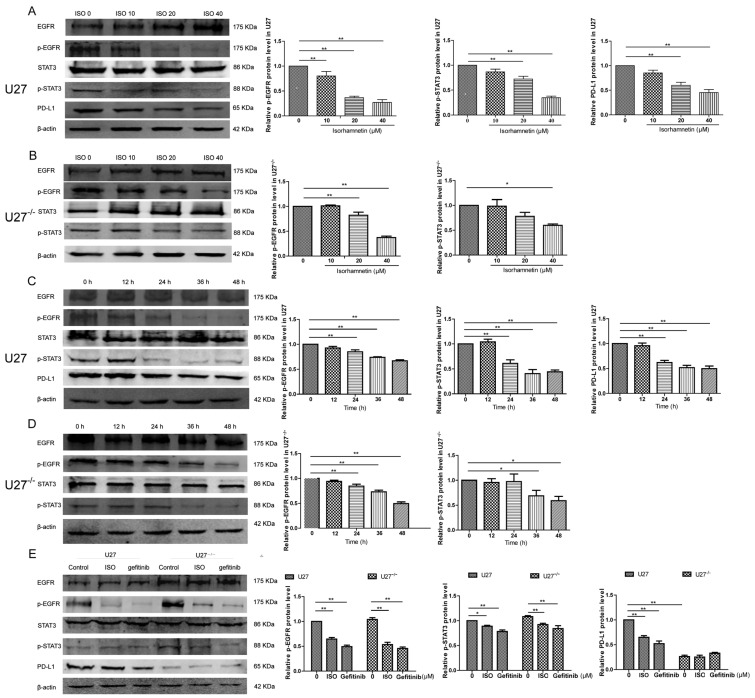
Effect of ISO on protein expression in U27 cells and U27^−/−^ cells. (**A**,**B**) p-EGFR, p-STAT3, and PD-L1 expression in (**A**) U27 and (**B**) U27^−/−^ cells with different concentrations of ISO. (**C**,**D**) p-EGFR, p-STAT3, and PD-L1 expression in (**C**) U27 and (**D**) U27^−/−^ cells exposed to ISO for different lengths of time. (**E**) p-EGFR, p-STAT3, and PD-L1 expression in U27 and U27^−/−^ cell exposed to either ISO or gefitinib. Data are mean ± SD. * *p* < 0.05; ** *p* < 0.01 on one-way ANOVA.

The expression of p-EGFR, p-STAT3, and PD-L1 in U27 and U27^−/−^ cells gradually decreased with the increase in ISO concentration (Figure 4A,B), along with the expression of p-EGFR and p-STAT3 in U27^−/−^ cells treated with 10 μM of ISO, which was not significantly different from the control group. ISO-treated U27 cells showed a change in expression of p-EGFR and p-STAT3 at 24 h (Figure 4C), whereas ISO-treated U27^−/−^ cells showed a change in expression of p-STAT3 at 36 h (Figure 4D). p-EGFR expression decreased in U27 and U27^−/−^ cells after the addition of gefitinib, and PD-L1 expression was significantly lower in U27^−/−^ cells than in U27 cells (*p* < 0.01), which suggested that ISO induces cell injury through the EGFR–STAT3–PD-L1 axis (Figure 4E).

### 2.4. ISO Inhibits Mammary Tumor Growth in U27 and U27^−/−^ Xenograft Mice

**Figure 5:**
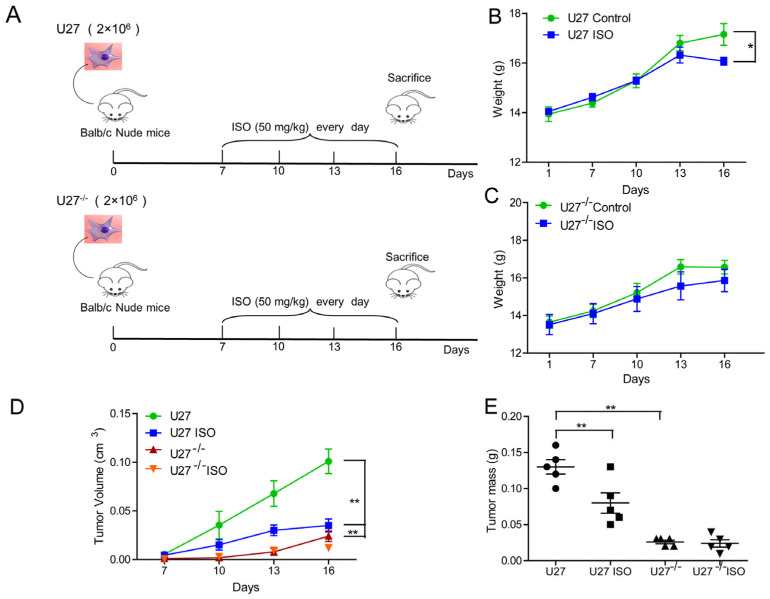
ISO inhibits tumor growth in U27 and U27^−/−^ xenograft mice. (**A**) Flow diagram of experimental procedure to inoculate female nude mice with U27 or U27^−/−^ cells followed by treatment intraperitoneally with 0.9% normal saline as control or ISO every 3 days until sacrifice (*n* = 5). (**B**) Murine body weight in control group mice and ISO group mice inoculated with U27 cells. (**C**) Murine body weight in control group mice and ISO group mice inoculated with U27^−/−^ cells. (**D**) Tumor growth curves for different treatment groups. (**E**) Tumor mass of control and ISO-treated groups. Data are mean ± SD. * *p* < 0.05; ** *p* < 0.01 on one-way ANOVA.

U27 and U27^−/−^ cells were inoculated subcutaneously in nude mice (Figure 5A). U27 cells underwent successful tumor loading in nude mice, and ISO inhibited the growth of mammary carcinoma in this group (Figure S5A,B). Tumors induced in U27^−/−^ cells were small in volume, and the effect of ISO treatment was not obvious (Figure S5C,D).

**Figure 6:**
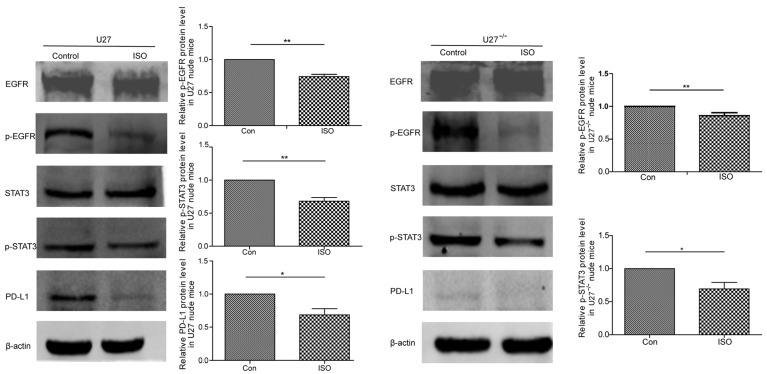
ISO inhibits EGFR-STAT3-PD-L1 signaling pathway. Western blot of EGFR, p-EGFR, STAT3, p-STAT3, and PD-L1 expression in mice inoculated with either U27 or U27^−/−^ cells and treated with ISO (50 mg/kg) (*n* = 3) compared with controls. Data are mean ± SD. * *p* < 0.05; ** *p* < 0.01 on one-way ANOVA.

The body weight (Figure 5B,C), tumor volume (Figure 5D), and tumor mass (Figure 5E) of ISO-treated U27 xenograft mice were all significantly lower compared with those of control mice. The mammary tumor volume of U27^−/−^ cell-bearing mice was significantly lower than that of the U27 group. Western blots showed that ISO significantly reduced the expression of p-EGFR, p-STAT3, and PD-L1 in tissues, which was consistent with the results in vitro (Figure 6). The tumor suppression rate of ISO was 46% in U27 cell-bearing mice compared with 7% in U27^−/−^ cell-bearing mice.

ImageJ graphic software (1.8.0) was used to quantify the protein in immunohistochemistry (IHC) images. ISO promoted caspase3 (Figure S6A), and inhibited Ki-67 (Figure S6B) and PD-L1 expression (Figure S6C) in U27 tumor-bearing mice. In the ISO-treated group, the expression rate of caspase3 increased from 20.14% to 30.40%, that of Ki-67 decreased from 24.74% to 7.20%, and that of PD-L1 decreased from 33.92% to 17.32%.

ISO treatment had no significant effect on caspase3 (Figure S6D), Ki-67 (Figure S6E), and PD-L1 expression (Figure S6F) in U27^−/−^ cell tumor-bearing mice. Caspase3 showed a positive expression rate from 10.32% to 10.26%, Ki-67 showed a positive expression rate from 13.18% to 18.66%, and PD-L1 showed a positive expression rate from 0.67% to 0.42%. These suggested that ISO inhibits mammary tumor growth by regulating PD-L1.

### 2.5. ISO Inhibits Tumor Growth in a Syngeneic Murine Mammary Tumor Model

**Figure 7:**
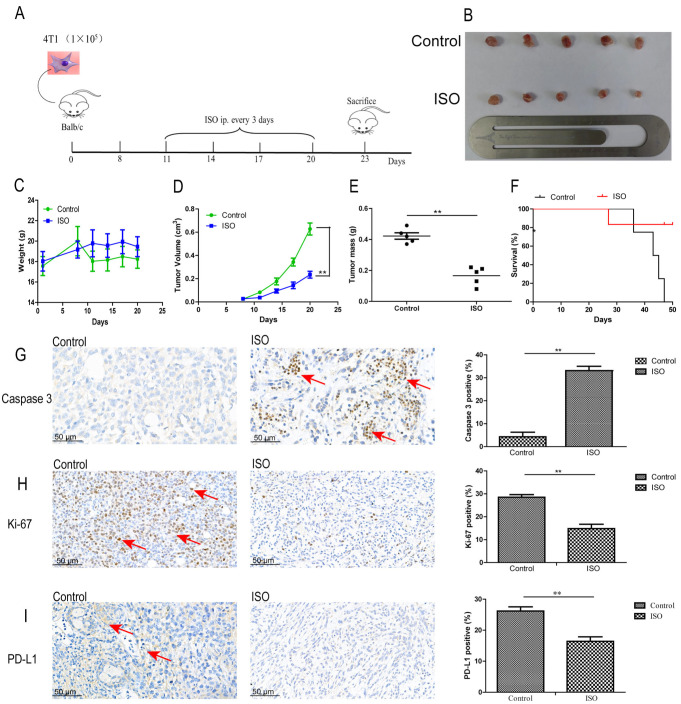
ISO inhibits tumor growth in an immunocompetent allograft model. (**A**) Flow diagram of experimental procedure to inoculate female BALB/c mice with 4T1 cells subcutaneously followed by treatment intraperitoneally with 0.9% normal saline as control or ISO every 3 days until sacrifice (*n* = 5). (**B**) Representative images of primary tumors. (**C**) Mouse body weight in different treatment groups. (**D**) Tumor growth curves for control group and ISO-treated mice. (**E**) Tumor mass of control and ISO-treated groups. Each black dots and squares in the picture represent a mice. (**F**) Overall survival rate of mice in different treatment groups (*n* = 6). Immunohistochemical of cells and quantification in 4T1-treated control and ISO-treated mice (400×) for (**G**) caspase3, (**H**) Ki-67, (**I**) PD-L1. Bar = 50 μm. Data are mean ± SD. ** *p* <0.01 on one-way ANOVA. The red arrows represent positive cells.

Mouse breast carcinoma cells 4T1 were used to establish a mammary cancer model in BALB/c tumor-bearing mice, which were then treated with ISO (Figure 7A). Mammary carcinomas were larger in the control group and reduced in the ISO group (Figure 7B). There was no difference in body weight between the groups (Figure 7C). The tumor volume (Figure 7D) and mass (Figure 7E) of mice in the ISO group were significantly lower than those in the control group. The tumor suppression rate was 61% in the ISO-treated group. The median survival of the ISO treatment group was 47 days (Figure 7F).

In immunohistochemistry (IHC) assays of the ISO-treated group, caspase3 (29.67% to 3.57%; Figure 7G), Ki-67 (28.5% to14.86%; Figure 7H), and PD-L1 (26.19% to 16.4%; Figure 7I) showed a positive expression rate.

ISO administration caused no toxicity to major organs when evaluated by hematoxylin and eosin staining (H & E) staining of the liver, heart, lung, spleen, and kidney (Figure S7).

### 2.6. ISO Directly Interacts with EGFR

**Figure 8:**
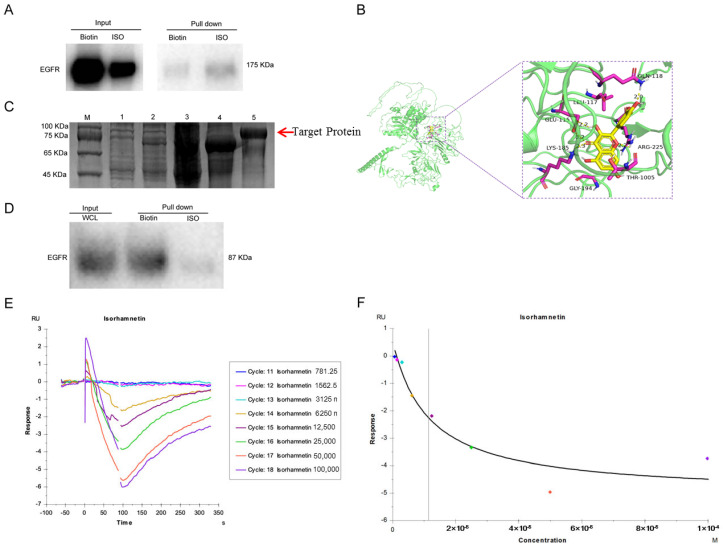
ISO direct binds EGFR. (**A**) Western blot with anti-EGFR antibodies of protein precipitated by streptavidin beads from U27 cell lysates in the presence of biotin or B-ISO (5 mM). (**B**) Representations of the predicted binding sites of ISO with EGFR. (**C**) Canine EGFR protein induction purification. M, Maker (GenStar M221); 1, EGFR/Bl21 bacteria before induction; 2, EGFR/Bl21 bacteria after induction (arrow indicates the target protein); 3, supernatant protein in bacterial fragmentation; 4, precipitate protein in bacterial fragmentation; 5, purified canine EGFR protein. (**D**) Western blot with anti-EGFR antibodies of purified proteins (His-EGFR) incubated with ISO (5 mM) precipitated by streptavidin beads. (**E**) ISO with purified EGFR protein in a multicycle dynamic test. (**F**) ISO with purified EGFR protein in an affinity fitting test. Different colored dots and gray vertical line means affinity fitting results of ISO with target protein EGFR.

To gain insights into how ISO affects PD-L1 expression, we confirmed the direct target proteins of ISO by purifying ISO-binding proteins from U27 cell lysates treated with ISO. We then incubated lysates of U27 cells with ISO, followed by affinity purification with streptavidin beads. The specific ISO-associated proteins were resolved by electrophoresis and identified by MS (Table S1). ISO could pull down EGFR protein from the lysates of U27 cells (Figure 8A). In addition, to determine whether ISO interacts with EGFR indirectly or directly, we performed a docking analysis of ISO in the built-in ligand-binding pocket of canine EGFR (XP_533073.3) by using the Libdock program in Discovery Studio 3.1 software (Figure 8B). The binding energy of ISO to EGFR protein was −7.4 kcal/mol, indicating a good binding effect. ISO interacted with the protein mainly via the formation of hydrogen bonds with ARG-225, GLN-118, GLU-115, and LYS-185, with hydrogen bond lengths of 2.2, 2.0, 2.2, 3.2, and 2.3 Å, respectively, and via hydrophobic interactions with THR-1005, GLY-194, and LEU-117.

To determine the direct interaction between ISO and EGFR, we purified the recombinant domain of EGFR fused with a His-tag, and the protein was successfully expressed at ~75 kDa (Figure 8C). In vitro pull-down assays showed that EGFR was pulled down with ISO (Figure 8D), suggesting that ISO directly binds EGFR. Furthermore, we examined the binding of ISO to EGFR by SPR, showing the affinity KD between EGFR and ISO to be 11.40 μM (Figure 8E,F). Collectively, our data suggest EGFR as a direct target of ISO.

The proteins obtained after pull-down were subjected to Gene Ontology (GO) and Kyoto Encyclopedia of Genes and Genomes (KEGG) analyses. GO analysis revealed that biological processes in the biotin-labeled ISO group of U27 cells were mainly involved in the organonitrogen compound biosynthetic process, whereas the molecular function was mainly involved in structural molecule activity. The main cellular components were the cytoplasm and endomembrane system. Kyoto Encyclopedia of Genes and Genomes (KEGG)-based analysis revealed that the biological functions of the biotin-labeled ISO group of U27 cells were mainly enriched in coronavirus disease and ribosome and protein processing in the endoplasmic reticulum (Figure S8).

## 3. Discussion

The increased life expectancy of dogs as a result of improved nutrition and vaccine use is one of the reasons for the high incidence of canine cancer [16]. Canine mammary tumors are the most common tumor in unsterilized female dogs, with an annual incidence of ~198/100,000 [17]. Cancer is also the health issue of greatest concern to dog owners [18].

There is a growing body of literature reporting how ISO, an active ingredient of traditional Chinese medicine, induces apoptosis in various human cancer cells [19]. By analyzing the active ingredients in Guiqi Baizhu, Ling et al. found that active ingredients, such as ISO, had potential therapeutic efficacy in cancer treatment [20]. However, no studies of the use of ISO against canine mammary tumors have been reported. Therefore, the current study analyzed the ability of ISO to induce canine mammary tumor cell damage. As expected, ISO showed robust inhibition of tumor growth in both nude mice and BALB/c mice, increasing the survival of tumor-bearing mice. In an in vivo trial, when U27^−/−^ cells were used in the same numbers as U27 cells to establish a mouse mammary cancer model, U27^−/−^-induced tumor growth was slower, which was consistent with the results of slow cancer growth in hormonal mice with LSD1 deletion [21]. In investigating the effect of ISO on BALB/c mice with mammary tumors, because the mice have an intact immune system, the anti-cancer effects in response to treatment with ISO were more prominent in terms of inhibiting PD-L1 and Ki-67 and promoting caspase3 expression, based on ICH results.

U27 cells have strong migratory and invasive abilities. The U27^−/−^ cells constructed with knockdown of PD-L1 exhibited slow cell growth and reduced cell migration and invasion ability. ISO inhibited the migration and invasion ability of U27 cells, but the sensitivity of U27^−/−^ cells to ISO was decreased, suggesting that ISO regulates PD-L1 expression in cells to induce cell damage. Interestingly, despite the knockdown of PD-L1 in U27^−/−^ cells, a shallow band was still detected on Western blot, similar to findings reported previously [22]. This might be because the primary antibody (only found in mice and rabbits) was not specific enough, and there is no monoclonal antibody specifically for canine PD-L1, an issue that needs to be resolved in future work. We have found that in many studies, the protein is still expressed at a low level after knockdown. This situation exists in a wide range; for example, after knocking down the gene in hepatitis B virus S gene [23], chicken liver cancer cell lines LMH *VNN1* gene [24], and early rat embryos *ISCA1* gene [25], the expression of knockdown proteins can still be detected by Western blot. This may be related to the efficiency of CRISPR/Cas9 knockdown, and we considered a 30% or more reduction in gene expression to be a successful knockdown. In this experiment, the gene expression after knockdown was 34.6% of the wild type, and we consider the knockdown to be successful.

The EGFR-STAT3 signaling pathway is involved in cancer development [26,27]. In addition, STAT3 is an important initiator of the expression of PD-L1 and has been shown to be involved in cancer proliferation [28,29]. In the present study, ISO induced U27 cell injury by downregulating the EGFR–STAT3–PD-L1 signaling pathway, consistent with the results of adding gefitinib (an EGFR inhibitor). In U27^−/−^ cells, the sensitivity of protein expression was reduced, consistent with the results of Lu et al. [10], indicating that ISO could inhibit the metastasis and growth of human colon cancer cells by modulating the PD-L1 pathway. Interestingly, we detected two phosphorylation sites of STAT3 (Ser727 and Tyr706), although only the phosphorylation signal of the former site was observed, indicating that ISO acts by phosphorylating Ser727, which was consistent with the findings of Zhang et al. [30] and Balic et al. [31].

Pull-down assay results of ISO-treated U27 cells showed that EGFR can bind to ISO, suggesting that ISO acts on EGFR to induce cell injury. Reports showed that ISO directly binds to estrogen receptor 1 (ESR1) to suppress ovarian cancer [32]. Li et al. reported that ISO inhibited gastric cancer by targeting HER-2 and PD-L1 [20]. We did not detect these target proteins in the current study, although EGFR is a very important biomarker in cancers. In addition, this result was consistent with the most enriched pathway in the GO and KEGG analyses.

SPR results again confirmed EGFR as the direct target of ISO. Interestingly, our results were negative SPR signals; we confirmed that the result was not produced by the nonspecific binding of the reference channel but rather by the test channel of the coupled protein after the action of ISO. For example, this might result from a change in the conformation of the protein after the action of ISO, leading to a change in its hydrated membrane morphology and a reduction in its mass, which was consistent with the findings of Bonnet et al. [33] and Pons et al. [34]. The recombinant canine EGFR comprises 629 amino acids and has a predicted molecular mass of 69.8 kDa. The apparent molecular mass of the protein was ~87.2 kDa in SDS-PAGE under reducing conditions. The reason for the larger molecular weight of the protein might be that urea breaks the hydrogen bonds and hydrophobic interactions between proteins, loosening the molecular structure [35].

## 4. Materials and Methods

### 4.1. Cell Culture

U27 (canine mammary tumor cells) and 4T1 (murine breast tumor cells) cell lines were cultured in Roswell Park Memorial Institute-1640 medium (Roswell Park Memorial Institute, (RPMI-1640), VivaCell, Shanghai, China) supplemented with 10% fetal bovine serum (FBS, VivaCell, Shanghai, China) and 1% penicillin/streptomycin (VivaCell). Cells were incubated at 37 °C in a humidified incubator containing 5% CO_2_.

Gefitinib (Med Chem Express (MCE), ZD1839, South Brunswick, NJ, USA), a potent EGFR inhibitor, was used to inhibit EGFR phosphorylation. In the control group, cells were treated with 0.1% DMSO, whereas the inhibitor group was treated with gefitinib (10 μM). Both co-culture treatments were maintained for 12 h.

### 4.2. CRISPR/Cas9-Mediated CD274 Knockout

Deletion of *CD274* in U27 cell lines was accomplished by Cyagen (Guangzhou, China). Briefly, the third exon gRNA of canine CD274 gene was designed and synthesized according to online websites “http://tools.genome-engineering.org (accessed on 20 April 2021)”. Sequence was synthesized by Beijing Liuhe Huada Gene Technology Co., Ltd. (Beijing, China). Then, gRNA concentration was adjusted to 2 μg/μL and stored at −20 °C. sgRNA (22.5 pmol), Cas9 protein (7.5 pmol), and phosphate-buffered saline (PBS) buffer were added in a DNase/RNase-free 1.5 mL centrifuge tube to a total volume of 7 μL. It was named RNP mixture and incubated at room temperature for 10 min. Next, HDRT (2 μg (dsDNA)) was added and incubated at room temperature for 2 min. Then, U27 cells (4 × 10^6^/μL) were mixed with the RNP mixture and performed electroporation on the Neon^TM^ electroporator (MPK5000, Invitrogen, Waltham, MA, USA). Electroporation parameters (for U27 cells voltage: 1200 V; time: 10 ms; pulse: 3 pulses). The completed electroporation sample was transferred to a 12-well plate, and one sample was placed into one well. Cells were incubated at a humidified CO_2_ incubator at 37 °C.

When cells were grown into clusters, the cells were digested with EDTA-free trypsin (Solarbio, Beijing, China) and inoculated in 96-well plates until there was only one cell in the well, which was a monoclonal well. The monoclonal wells were cultivated for 14 days. Monoclonal cells with CRISPR/Cas9-mediated *CD274* deletion were obtained using PCR (Table S2). Finally, Sanger sequencing and Western Blot were used to verify the homozygotes (Figure S1).

### 4.3. Chemical Concentration Screening

ISO was purchased from Shanghai Tauto Biotech Co., Ltd. (Shanghai, China) with a purity of ≥98%. ISO was aliquoted at a concentration of 30 mM in DMSO and stored at −20 °C until use. Drug concentration screening was estimated by using the CCK-8 assay (CK001-500T, LABLEAD Inc., Beijing, China). Briefly, 10^4^ cells were seeded in 96-well plates with 100 μL RPMI-1640 medium in each well. After 24 h cultivation, 0, 40, 80, 160, and 320 μM of ISO in basic medium were added. After 48 h of co-culture, each well was incubated with 10 μL CCK-8 solution for 2 h away from light before measuring the absorbance at 450 nm using a PerkinElmer’s EnSpire Multilabel Plate Reader (PerkinElmer, Wellesley, MA, USA). ISO-biotin (B-ISO) was synthesized by Wayen Biotechnologies (Shanghai, China) for pull-down assays (Figure S2).

### 4.4. Cell Viability Assay

To investigate their growth velocity, U27 and U27^−/−^ cells were cultured in 6-well plates at a density of 5 × 10^3^ cells per well. After 48 h of incubation, cells from three wells were removed, digested with trypsin, and then centrifuged at 1000× *g* for 10 min. The supernatant was then removed, and fresh medium was added to resuspend the cells to form a single-cell suspension. Then, 10 μL of cell suspension was absorbed and added to a blood cell counting plate for counting under a microscope (CKX41, Olympus Corporation, Tokyo, Japan). Samples were taken every 24 h for 6 days, and a cell growth curve was plotted with the resulting data. The experiment was performed in triplicate.

### 4.5. Migration and Invasion Assays

CMT-U27 cells (2 × 10^3^ cells/insert) were seeded into the upper chamber (8 μm, Corning Inc., Corning, NY, USA) of 24-well Transwell plates (Corning) in media containing different concentrations of ISO (0, 10, 20, and 40 µM). For the invasion assay, the upper chambers were coated with Matrigel (BD Biosciences, San Jose, CA, USA), and 600 µL of complete medium was added to the lower chambers. After incubating for 48 h, the initial medium and Matrigel were discarded. The cells that settled at the bottom of the chamber were monitored after Crystal Violet staining for 3 min and washing with PBS. Five fields per well were randomly chosen, and the cells were counted using an inverted microscope at 20× magnification (Olympus IX53/DP80, Tokyo, Japan). The experiment was performed in triplicate.

### 4.6. TUNEL Assay

For TUNEL assays, to determine ISO-induced cell apoptosis, CMT-U27 cells (2 × 10^3^ cells/insert) were seeded into 24-well Transwell plates (Corning) in media containing either different concentrations of ISO (0, 10, 20, and 40 µM) for 48 h or gefitinib (10 µM) for 12 h. The cells were fixed with 4% paraformaldehyde for 30 min, and then PBS was added containing 0.3% Triton X-100. The TUNEL detection solution was then incubated in the dark for 60 min at 37 °C. The cells were washed three times with PBS, after which the plates were sealed with anti-fluorescence burst sealing solution and observed under a fluorescence microscope (CKX41, Olympus Corporation, Tokyo, Japan).

### 4.7. JC-1 Assay

For JC-1 assays, to investigate whether ISO impacted the mitochondrial membrane potential, CMT-U27 cells (2 × 10^3^ cells/insert) were seeded into 6-well Transwell plates (Corning) in media containing either different concentrations of ISO (0, 10, 20, and 40 µM) for 48 h or gefitinib (10 µM) for 12 h. CCCP (10 mM) provided in the JC-1 kit (C2003S, Beyotime, Shanghai, China) was used to dilute the cell culture medium to 10 µM, and the cells were left for 20 min as a positive control. The cells were incubated for 20 min at 37 °C in a cell incubator before being washed twice with JC-1 staining buffer. Finally, 2 mL of cell culture solution was added to a cell incubator, and cells were observed under a fluorescence microscope.

### 4.8. Immunofluorescence Assay

Polyline (Solarbio, P8141) pretreated coverslips, which were embedded in 24-well plates, were seeded with U27 cells at a density of 1 × 10^4^ cells per well. PD-L1 was diluted to 1 µg/mL as the primary antibody (Thermo Fisher Scientific, Waltham, MA, USA; 14-5983-82). After treatment with different concentrations of ISO (0, 10, 20, and 40 µM) for 48 h, cells were treated with acetone:methanol (1:1) for 20 min. PD-L1 was used to stain cells for 16 h at 4 °C. Cells were then washed with PBS and cultured with secondary antibodies (1 µg/mL FITC goat anti-mouse IgG; Abcam, Cambridge, UK; ab6785) for 1 h. Finally, cells were washed with PBS and incubated with DAPI (BD Transduction Laboratories, Franklin Lakes, NJ, USA) and then examined under a fluorescence microscope (CKX41).

### 4.9. Annexin V-FITC/PI Double Staining Assay

U27 cells (2 × 10^5^ cells/well/mL) were seeded in 6-well plates, and basic medium containing ISO (20 and 40 μM) was added to each well. Annexin V-FITC/PI Apoptosis Detection Kit (Invitrogen, USA)was used to detect the level of cell apoptosis. After 48 h, the medium was collected, and adherent cells were removed using EDTA-free trypsin (Solarbio, Beijing, China). Samples were centrifuged at 1000× *g* for 5 min at 4 °C. Next, samples were incubated with Annexin V-FITC solution in the dark for 15 min at 25 °C. Then, propidium iodine (PI) solution was added and incubated for 5 min in the dark. FACSVerse flow cytometer25 (FCM, BD Biosciences, San Jose, CA, USA) was used to analyze the number of apoptotic cells, and FlowJo v10.7 software (Tree Star, Woodburn, OR, USA) was used to determine the apoptotic ratio. Quadrant 1 represents early apoptosis, quadrant 2 represents late apoptotic cells, quadrant 3 represents necrotic cells, and quadrant 4 represents live cells. The apoptosis rate was calculated as the sum of the percentages of cells in quadrant 1 section and quadrant 2 section.

### 4.10. Animal Studies

All animal procedures were performed following the Guidelines for Care and Use of Laboratory Animals of Beijing Municipal Academy of Agriculture and Forestry and approved by the Animal Care and Use Committee of the Institute of Animal Husbandry and Veterinary Medicine (approval number: IHVM11-2302-5).

For murine xenotransplantation models, a cell suspension of 10^7^ U27 cells or 10^7^ U27^−/−^ cells in 0.2 mL PBS was inoculated subcutaneously into the left mammary fat pads of 20 5-week-old female BALB/SCID nude mice. Tumors were visible after ~7 days. Mice were randomly separated into the control group (0.9% normal saline) and ISO group (50 mg/kg); the latter were injected with ISO three times a day for nine days.

To develop murine immunocompetent allograft mammary tumor models, a cell suspension of 10^5^ 4T1 cells in 0.1 mL PBS was inoculated subcutaneously into the left mammary fat pads of 10 5-week-old female BALB/c mice. Once the tumor was visible, the ISO group was injected with 50 mg/kg ISO intraperitoneally every 3 days for 12 days, and the control group was injected with 0.9% normal saline. Body weight and tumor volume were measured every 2 days. Tumor volume was determined by using Equation (1):

V = largest diameter × smallest diameter^2^/2 (1)

At the end of the experiment, the mice were sacrificed, and each group of tumors was excised, weighed, and prepared for further experiments.

For the survival assay, 4T1 cells (1 × 10^4^) were injected into the left fat pad of 12 5-week-old female BALB/c mice. Once tumors were visible, mice were randomly separated into two groups (six mice/group) and received intraperitoneal administration of ISO (50 mg/kg) or 0.9% normal saline every 3 days. The mice were observed daily until their death. The experiment ended once all the mice in the control group had died. One-way analysis of variance (ANOVA) was used for comparisons between different groups.

### 4.11. Western Blotting

U27 and U27^−/−^ cells (5 × 10^5^ cells) were treated with different concentrations of ISO (0, 10, 20, and 40 µM) for 48 h and then used for protein extraction. Whole-protein extraction of U27, U27^−/−^, and tumor tissues from sacrificed mice was performed using RIPA lysis buffer (MCE, HY-K1001) and a 10% protease inhibitor cocktail (MCE, HY-K0010). A BCA Protein Assay Kit (Beyotime, P0012) was used to determine protein concentrations. SDS-PAGE was used to separate lysate proteins and transfer proteins to PVDF membranes, which were then incubated with primary antibodies against EGFR (1:1000, Cell Signaling Technology, Danvers, MA, USA; #4267), p-EGFR (1:1000, Cell Signaling Technology, #3777T), STAT3 (1:1000, Cell Signaling Technology, #9139), p-STAT3(1:1000, phosphor S727, Abcam, ab30647), p-STAT3 (1:1000, phosphor Y705, ab76315), PD-L1 (1:1000, Thermo Fisher Scientific, 14-5983-82), and β-actin (1:1000, Invitrogen, MA1-140) [36] overnight at 4 °C. Primary antibodies were diluted in a ratio of 1:1000. Secondary antibodies were IRDye 800CW goat anti-mouse IgG (H + L), (926-32210, LI-COR) or IRDye 800CW goat anti-rabbit IgG (H + L), (926-32211, LI-COR) at a dilution of 1:15,000, and membranes were incubated with the secondary antibodies for 1 h. The antigens were then visualized with the Odyssey infrared imaging system (LI-COR Biosciences, Lincoln, NE, USA). β-actin was used as a loading control. The relative density of protein bands in each blot was measured using the ImageJ program (version 1.51j8, National Institutes of Health, Bethesda, MD, USA).

### 4.12. Drug Toxicity Assay

4T1 was inoculated into ten 5-week-old female BALB/c mice subcutaneously, as described in Section 4.10 After injection with ISO (100 mg/kg) or 0.9% normal saline for 20 days, BALB/c mice were sacrificed. The organs were dissected, fixed in 4% paraformaldehyde, and then dehydrated and embedded in paraffin. Samples were then cut into 4 µm thick sections and stained with hematoxylin and eosin (H&E). The slides were observed under a light microscope (Nikon, Tokyo, Japan).

### 4.13. Immunohistochemical Staining

For immunohistochemical (IHC) analysis, U27 cell xenograft tumors, U27^−/−^ cell xenograft tumors, and 4T1-induced tumors were prepared in 3 µm samples. Tumors were assessed with primary antibodies at a concentration of 1 µg/mL for Ki-67 (Abcam, ab16667), cleaved caspase3 (Cell Signaling Technology, # 9661), and PD-L1 (Thermo Fisher Scientific, 14-5983-82). Deparaffinized samples were placed in a PT module with EDTA buffer solution (pH 8.0) (Master Diagnostica, São Paulo, Brazil; MAD-004072R/D). The specimens were incubated with primary antibodies for 16 h at 4 °C and with secondary antibodies (ZSGB-BIO, Beijing, China) for 1 h at room temperature. Subsequently, 3,3′-diaminobenzidine tetrahydrochloride (ZSGB-BIO, China) was used to visualize antibody binding. Sections were observed under a light microscope (Olympus), and the number of positive cells was quantified by ImageJ software. The sample was considered positive for ki-67, cleaved caspase3, and PD-L1 if >10% of tumor cells were positive for antibody binding.

### 4.14. Construction of EGFR Expression Vectors

The sequence encoding the EGFR domain (amino acid residues Met1–Ser618) from domestic dog *Canis lupus familiaris* was cloned into the NdeI and XhoI sites of pET-28a (+) plasmids (GE Healthcare, Boston, MA, USA). Plasmids were then transfected into BL21 cells named Pet28a-EGFR-Bl21 (CWBIO, Beijing, China). Expression of His-EGFR recombinant protein was induced by isopropyl-β-D-thiogalactopyranoside for 4 h at 37 °C.

### 4.15. Protein Purification

Under ice bath conditions, Pet28a-EGFR-Bl21 bacteria were broken by ultrasonic waves (power 60%, ultrasound 5 s, rest 5 s, 30 min). The solution was then centrifuged at 12,000× *g* for 30 min, and the supernatant was then collected and precipitated. SDS-Page electrophoresis was performed by adding SDS loading buffer.

The bacterial precipitate recovered after ultrasonic centrifugation was washed with 2M urea and then 1% TritonX-100 (0.1 M PBS, pH 7.4) three times. Inclusion bodies were incubated with 8M urea on ice overnight so that they dissolved completely. The resulting solution was centrifuged at 12,000× *g* for 30 min to remove any insoluble impurities. The remaining protein was collected and stored at −80 °C until use. Proteins were quantified with the BCA protein assay kit (Pierce, Rockford, Rockford, IL, USA).

### 4.16. Predicted Binding Sites of EGFR and ISO

The 3D structure of ISO in SDF format was downloaded from PubChem and then imported into ChemBio3D Ultra 14.0 for energy minimization. The minimum RMS gradient was set to 0.001, and the small molecule was saved as a mol2 file. The optimized small molecule was then imported into AutodockTools-1.5.6 for hydrogenation, charge calculation, charge assignment, and rotatable key setting and saved as a “pdbqt” file. Then, the protein was imported into Pymol 2.3.0 to remove protein crystalline water and original ligands. The EGFR structure was imported into AutoDocktools (v1.5.6) for hydrogenation, charge calculation, charge assignment, and specification of atom types and was then saved as a “pdbqt” file. The protein-binding sites were predicted using POCASA 1.1 and docked using AutoDock Vina1.1.2 with protein-related parameters set to the following: center_x = −5.3; center_y = −0.3; center_z = 9.6; search space: size_x: 60; size_y: 60; size_z: 60 (the spacing of each grid point was 0.375 Å), and exhaustiveness: 10; the remaining parameters were set by default. 

### 4.17. Streptavidin–Biotin Affinity Pull-Down Assay

Cell lysis buffer containing a 1% protease inhibitor cocktail was used to lyse CMT-U27 cells. The cell lysates were cultivated with free biotin (MedChemExpress, MCE, South Brunswick, NJ, USA) or ISO-biotin (5 mM) for 5 h at 4 °C. Then, prewashed streptavidin agarose beads (Yeasen Biotech, Shanghai, China) were added and incubated overnight at 4 °C with rotation. Elution buffer was used to wash the beads three times, and then denatured protein was detected by SDS-PAGE and enzymes by trypsin overnight at 37 °C. After the enzymatic polypeptides were collected, the proteins were analyzed using mass spectrometry (MS, BGI, Beijing, China).

The differential proteins obtained from the comparison of control and ISO groups were subjected to Gene Ontology (GO) and Kyoto Encyclopedia of Genes and Genomes (KEGG) Pathway analyses. These were performed at two levels (bioinformatic annotation and analysis) to better understand the functional properties of these proteins and their relevance to the study target so that more relevant proteins could be selected for further research.

### 4.18. ISO and EGFR Surface Plasmon Resonance Assay

Sensor NTA chip was used to immobilize histidine-tagged proteins for surface plasmon resonance (SPR) assays. To do so, 5 mM NiCl_2_ flowed through the surface of the chip test channel at a flow rate of 30 μL/min for 120 s. After the target EGFR was captured by the divalent nickel ion in the analysis buffer, the protein was injected into the chip and was diluted to 20 μg/mL. The flow rate was set to 10 μL/min for 240 s to capture EGFR protein.

The binding properties of EGFR to ISO were initially determined and evaluated manually. The highest concentration of EGFR used was 100 μM, and nine concentrations were established to form a twofold gradient dilution: 0, 0.78125, 1.5625, 3.125, 6.25, 12.5, 25, 50, and 100 μM. The experiments were run in multiple cycles. The data were fitted using BIAcore T200 analysis software v3.2 (GE Biacore, Boston, MA, USA) using a 1:1 Langmuir binding model, and the steady-state fitting methods were used to determine the KD value.

### 4.19. Statistical Analysis

All data were expressed by GraphPad Prism 8.0 (San Diego, CA, USA) and are shown as means ± standard deviation (SD). An unpaired Student’s *t*-test and one-way analysis of variance (ANOVA) were used for comparisons between two different groups or larger numbers of groups. A log-rank test was used for the contrast of survival curves. *p*-values < 0.05 were considered to be statistically significant.

## 5. Conclusions

ISO inhibits CMT U27 cell migration and invasion and induces cell apoptosis. Knockdown of *CD274* decreased cell sensitivity and increased resistance of U27 cells to ISO. ISO exerts its effects by inhibiting cell surface PD-L1 expression and EGFR-STAT3-PD-L1 pathway expression by targeting EGFR to induce cell damage. ISO showed positive treatment effects on CMT and murine tumors. In addition, ISO inhibited tumor growth and promoted apoptosis of cancer cells. Such evidence provides theoretical support for the study of the mechanism of action of ISO and the development of new drugs for the treatment of canine breast cancer and is of great significance for the development of Chinese (veterinary) medicine.

## Data Availability

The datasets used and/or analyzed during the current study are available from the corresponding author upon reasonable request.

## Supplementary Materials

The supporting information can be downloaded at: https://www.mdpi.com/article/10.3390/ijms25010670/s1.

## Abbreviations

ISO: Isorhamnetin; MS: mass spectrometry; EGFR: epidermal growth factor receptor; PD-L1: programmed death ligand-1; RPMI-1640: Roswell Park Memorial Institute-1640; B-ISO: ISO-biotin; IHC: immunohistochemical staining; SPR: surface plasmon resonance; ANOVA: one-way analysis of variance; ESR1: estrogen receptor 1; GO: Gene Ontology; KEGG: Kyoto Encyclopedia of Genes and Genomes.
