# Supplementary material for: Isorhamnetin Regulates Programmed Death Ligand-1 Expression by Suppressing the EGFR–STAT3 Signaling Pathway in Canine Mammary Tumors
Source: Int J Mol Sci. 2024 Jan 4;25(1):670. doi: 10.3390/ijms25010670 (PMC10779303; doi:10.3390/ijms25010670)
Supplement: Supplementary file 1 [file ijms-25-00670-s001.zip › ijms-2681848-supplementary.pdf]

Supplementary Figures and legends

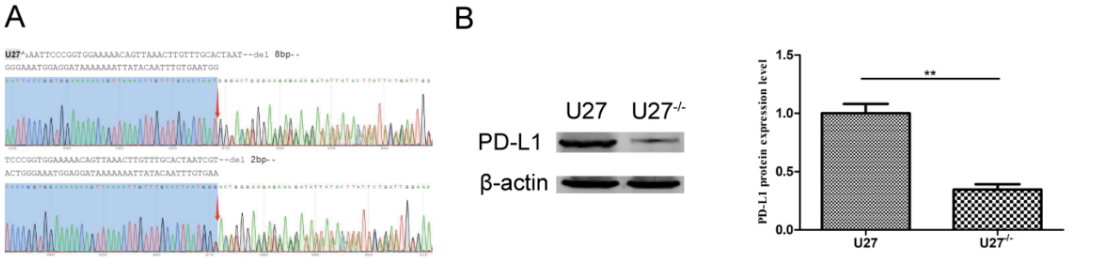


**Figure S1.** Nucleotide sequencing of *CD274* in $U27^{-/-}$ and PD-L1 expression in U27 and $U27^{-/-}$ cell. (**A**) Sequence determination of Knockout *CD274* in $U27^{-/-}$ cell. (**B**) PD-L1 expression in U27 and $U27^{-/-}$ cell. Data are mean ±SD. (n = 3, ** $p < 0.01$ on t-test)

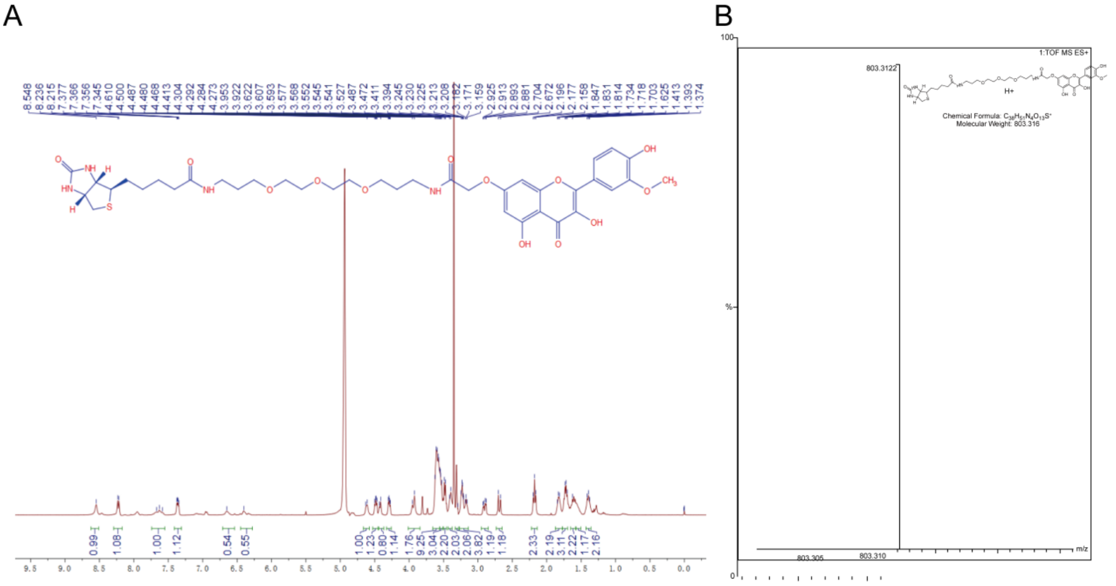


**Figure S2. (A) 1H NMR of ISO-PROTAC linker 24-biotin.**[1] H NMR (400 MHz, Methanol-d 4 ) δ 8.55 (s, 1H), 8.23 (d, J = 8.4 Hz, 1H), 7.63(t, J = 16.8 Hz, 1H), 7.36 (dd, J = 8.4, 4.2 Hz, 1H), 6.65 (s, 1H), 6.40 (s, 1H),4.62 (d, J = 7.8 Hz, 1H), 4.48 (dd, J = 7.7, 5.0 Hz, 1H), 4.42 (d, J = 7.3 Hz, 1H),4.29 (dd, J = 7.9, 4.4 Hz, 1H), 3.94 (d, J = 12.4 Hz, 2H), 3.66 – 3.56 (m, 9H), 3.55 – 3.51 (m, 3H), 3.48 (d, J = 6.0 Hz, 2H), 3.40 (d, J = 7.0 Hz, 2H), 3.29 (s, 2H), 3.27 – 3.14 (m, 4H), 2.90 (dd, J = 12.9, 4.8 Hz, 1H), 2.69 (d, J = 12.7 Hz, 1H), 2.18 (t, J = 7.4 Hz, 2H), 1.83 (t, J = 6.4 Hz, 2H), 1.77 – 1.70 (m, 3H), 1.60(d, J = 16.9 Hz, 2H), 1.39 (t, J = 7.8 Hz, 2H). **(B) Ion to mass charge ratio (m/z) mapping.** m/z calcd for $C_{38}H_{51}N_4O_{13}S^+$ 803.3168, found: 803.3122.

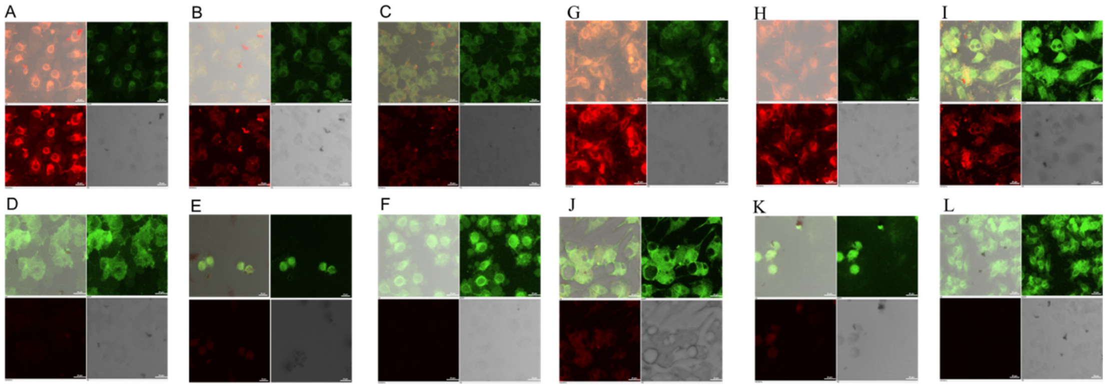


**Figure S3. ISO induces apoptosis by decreasing the mitochondrial membrane potential in U27 (A–F) and U27$^{-/-}$cells (G–L). (A,G)** control group; cells treated with 10 μM ISO **(B,H)**, 20 μM ISO **(C,I)**, 40 μM ISO **(D,J)**, gefitinib (10 μM) **(E,K)**, or CCCP **(F,L)**. Increasing drug concentration resulted in increased green fluorescence and cellular damage.

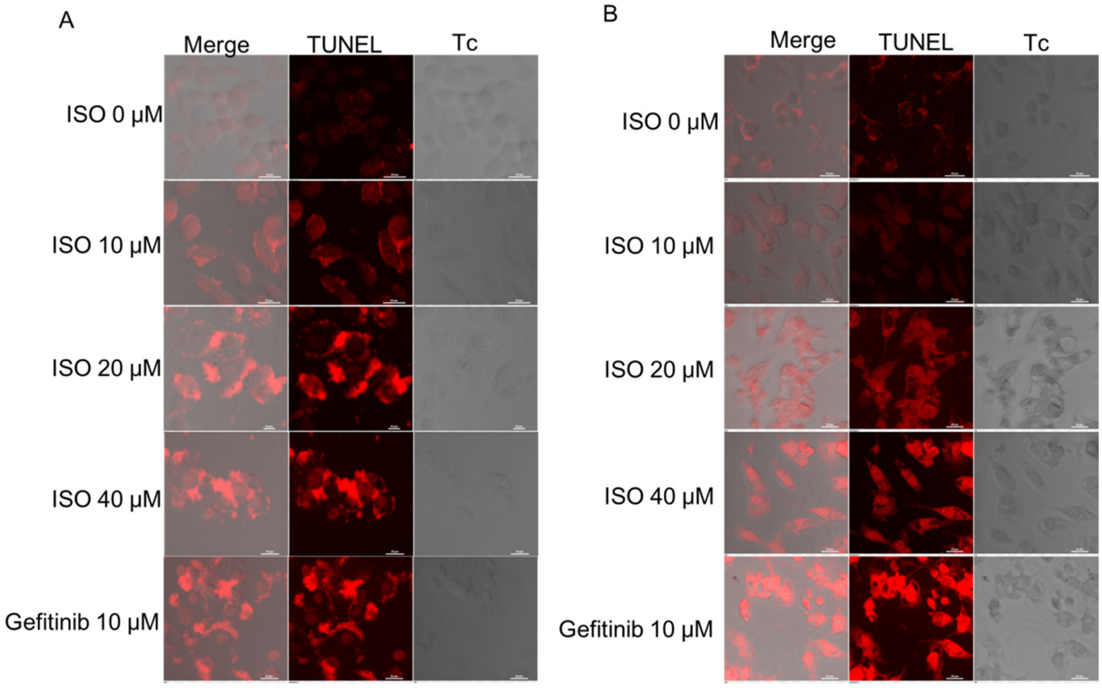


**Figure S4. Detection of ISO-induced apoptosis in U27(A) and U27$^{-/-}$ (B) cells by TUNEL assays.** ISO induced apoptosis of U27 cells along a concentration gradient **(A)** and reduced apoptosis in U27$^{-/-}$ cells **(B)**. With the increase in drug concentration, both the red fluorescence and degree of apoptosis of cells increased.

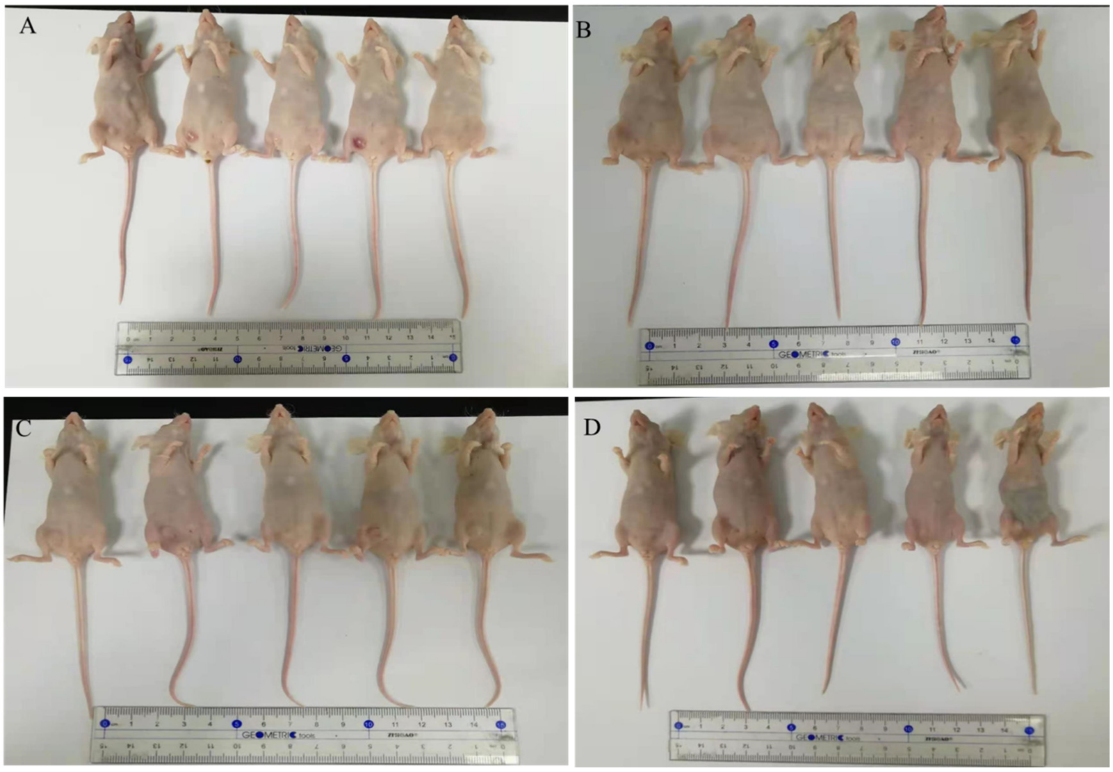


**Figure S5. ISO inhibits mammary tumor growth in U27 and U27$^{-/-}$ xenograft mice. (A)** control group with U27 treated. **(B)** ISO (50mg/kg) group with U27 treated. **(C)** control group with U27$^{-/-}$ treated. **(D)** ISO (50mg/kg) group with U27$^{-/-}$ treated.

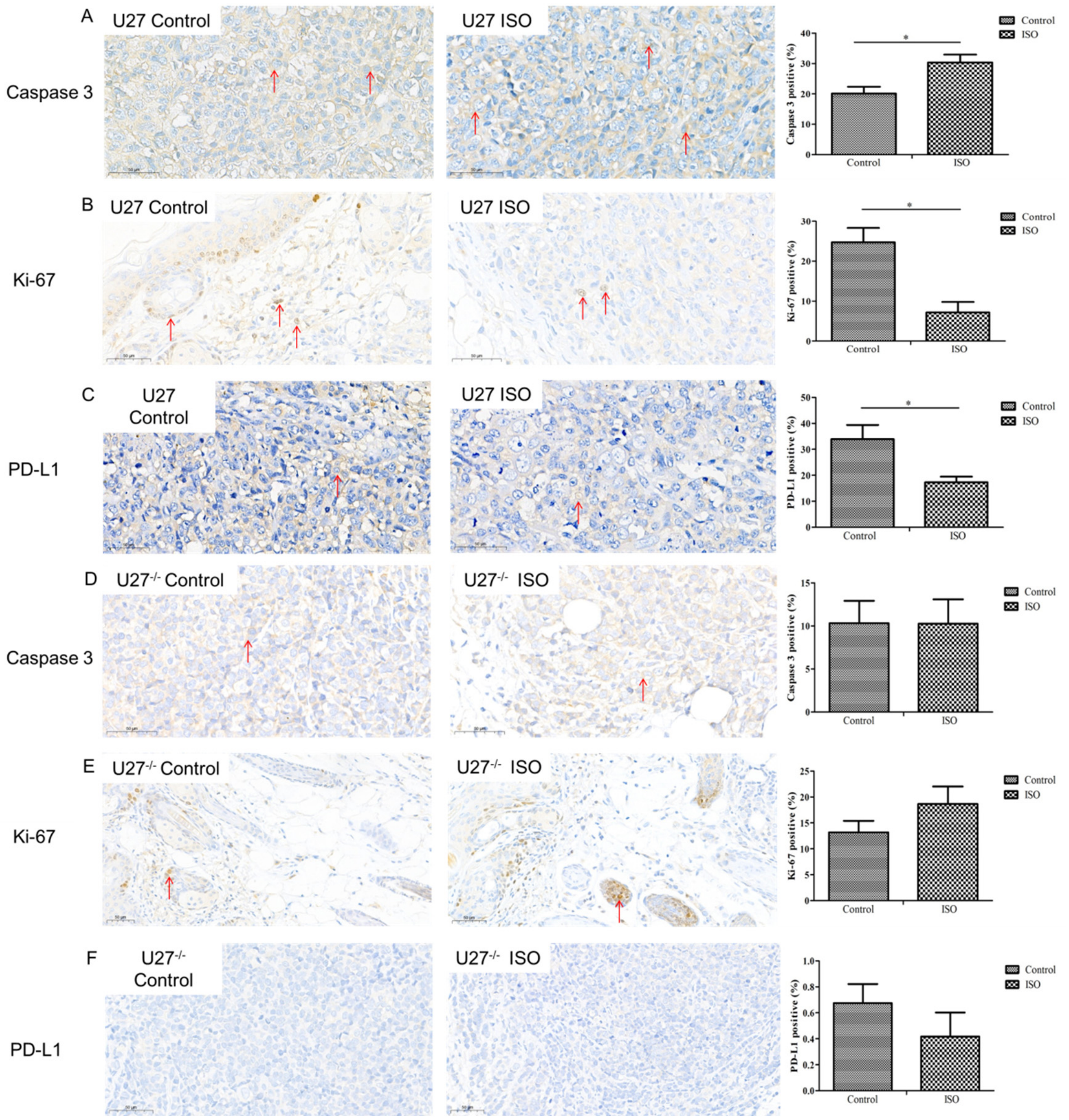


**Figure S6.** Immunohistochemical of cells and quantification in U27-treated control and ISO-treated mice (400X) and in U27$^{-/-}$-treated control and ISO-treated mice tissues for **(A, D)**, Caspase3, **(B, E)** Ki-67, **(C, F)** PD-L1.

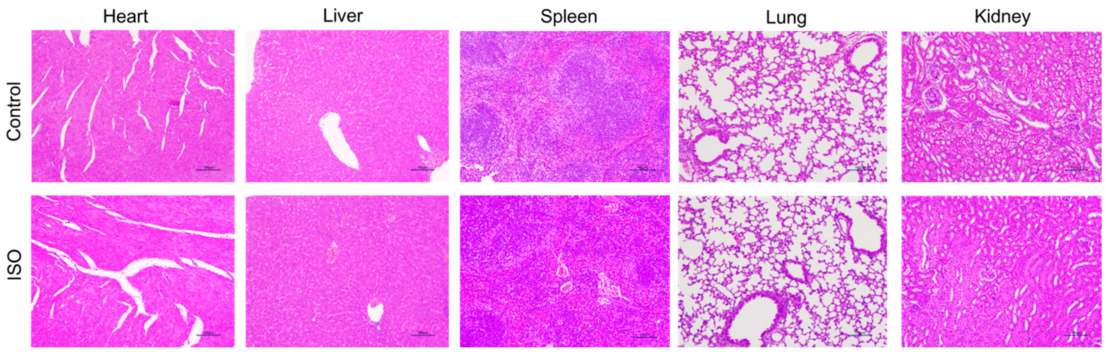


**Figure S7.** ISO shows low toxicity in a mouse model. H&E staining of various organs from control and ISO-treated mice (n=3).

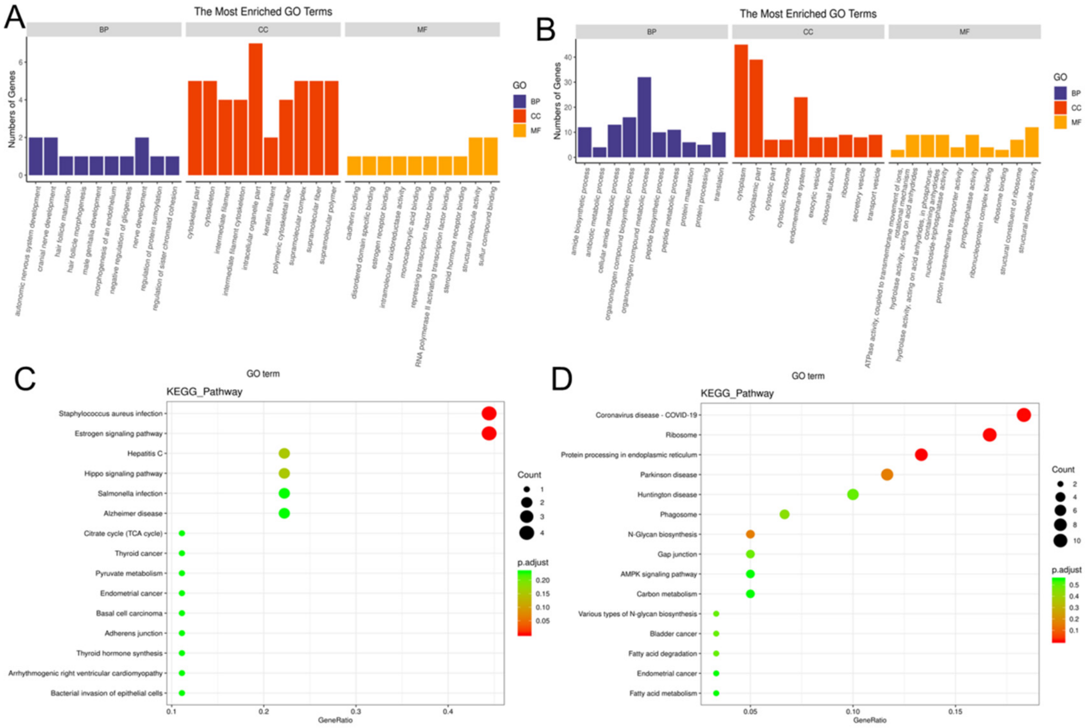


**Figure S8. GO and KEGG analyses.** GO analysis of the **(A)** bio-control group and **(B)** bio-ISO group. KEGG analysis of the **(C)** bio-control group and **(D)** bio-ISO group.

**Table S1.** Mass spectrometric detection of target bands.

| Accession | Gene Symbol | Description | Coverage | Peptides | PSMs | Unique Peptides | AAs | MW |
|---|---|---|---|---|---|---|---|---|
| L7N071 | ACTN4 | Actinin alpha 4 OS=Canis lupus familiaris OX=9615 GN=ACTN4 PE=3 SV=2 | 16 | 13 | 13 | 13 | 1163 | 131.6 |
| F1PIC7 | HSPA5 | 78 kDa glucose-regulated protein OS=Canis lupus familiaris OX=9615 GN=HSPA5 PE=3 SV=2 | 25 | 14 | 14 | 12 | 654 | 72.2 |
| F1PHQ0 | CLTC | Clathrin heavy chain OS=Canis lupus familiaris OX=9615 GN=CLTC PE=3 SV=3 | 10 | 12 | 12 | 12 | 1682 | 192.3 |
| F1P8N6 | HSP90B1 | Endoplasmin OS=Canis lupus familiaris OX=9615 GN=HSP90B1 PE=3 SV=1 | 14 | 10 | 10 | 8 | 803 | 92.3 |
| E2RNG2 | ATP5F1A | ATP synthase subunit alpha OS=Canis lupus familiaris OX=9615 GN=ATP5F1A PE=3 SV=2 | 16 | 8 | 8 | 8 | 605 | 64.6 |
| F6X6K8 | LRPPRC | Leucine rich pentatricopeptide repeat containing OS=Canis lupus familiaris OX=9615 GN=LRPPRC PE=4 SV=2 | 7 | 8 | 8 | 8 | 1393 | 157.6 |

| | | | | | | | | |
|---|---|---|---|---|---|---|---|---|
| F1PYU9 | KRT10 | Keratin, type I cytoskeletal 10 OS=Canis lupus familiaris OX=9615 GN=KRT10 PE=3 SV=2 | 11 | 8 | 8 | 7 | 568 | 57.7 |
| F1Q0B0 | NCL | Nucleolin OS=Canis lupus familiaris OX=9615 GN=NCL PE=4 SV=2 | 10 | 7 | 7 | 7 | 715 | 77.4 |
| A0A5F4CAZ7 | ATP1A1 | Sodium/potassium-transporting ATPase subunit alpha OS=Canis lupus familiaris OX=9615 GN=ATP1A1 PE=3 SV=1 | 8 | 7 | 7 | 7 | 1028 | 113.8 |
| E2R0T6 | HSPA8 | Heat shock protein family A (Hsp70) member 8 OS=Canis lupus familiaris OX=9615 GN=HSPA8 PE=3 SV=1 | 15 | 8 | 8 | 6 | 646 | 70.9 |
| Q6EIY9 | KRT1 | Keratin, type II cytoskeletal 1 OS=Canis lupus familiaris OX=9615 GN=KRT1 PE=2 SV=1 | 8 | 6 | 6 | 6 | 619 | 63.8 |
| F1PBJ3 | LMNA | Lamin A/C OS=Canis lupus familiaris OX=9615 GN=LMNA PE=3 SV=2 | 10 | 6 | 6 | 6 | 751 | 82.7 |
| A0A5F4D8X1 | HDLBP | High density lipoprotein binding protein OS=Canis lupus familiaris OX=9615 GN=HDLBP PE=4 SV=1 | 5 | 5 | 6 | 5 | 1569 | 171.2 |
| A0A5S6CY29 | ACTB | Actin, cytoplasmic 1 OS=Canis lupus familiaris OX=9615 GN=ACTB PE=3 SV=1 | 21 | 5 | 5 | 5 | 400 | 44.5 |
| F1PAR8 | PHB | Prohibitin OS=Canis lupus familiaris OX=9615 GN=PHB PE=3 SV=2 | 22 | 5 | 5 | 5 | 262 | 28.9 |
| A0A5F4CW76 | ATP5F1B | ATP synthase subunit beta OS=Canis lupus familiaris OX=9615 GN=ATP5F1B PE=3 SV=1 | 11 | 5 | 5 | 5 | 640 | 70.2 |
| A0A140T8E6 | JUP | Junction plakoglobin OS=Canis lupus familiaris OX=9615 GN=JUP PE=3 SV=1 | 8 | 5 | 5 | 5 | 744 | 81.7 |
| A0A5F4BS50 | MTHFD1L | Formyltetrahydrofolate synthetase OS=Canis lupus familiaris OX=9615 GN=MTHFD1L PE=3 SV=1 | 6 | 5 | 5 | 5 | 908 | 98.1 |
| E2RSI6 | EZR | Ezrin OS=Canis lupus familiaris OX=9615 GN=EZR PE=4 SV=1 | 7 | 4 | 5 | 4 | 586 | 69.4 |

| | | | | | | | | |
|---|---|---|---|---|---|---|---|---|
| E2QUU5 | HSPD1 | 60 kDa chaperonin OS=Canis lupus familiaris OX=9615 GN=HSPD1 PE=3 SV=2 | 9 | 4 | 4 | 4 | 573 | 61 |
| J9NXT1 | LOC608051 | Tubulin alpha chain OS=Canis lupus familiaris OX=9615 GN=LOC608051 PE=3 SV=1 | 12 | 4 | 4 | 4 | 450 | 49.9 |
| L7N095 | KRT75 | Keratin 75 OS=Canis lupus familiaris OX=9615 GN=KRT75 PE=3 SV=2 | 6 | 4 | 4 | 4 | 565 | 60.2 |
| A0A5F4CR13 | COPA | Coatomer subunit alpha OS=Canis lupus familiaris OX=9615 GN=COPA PE=4 SV=1 | 4 | 4 | 4 | 4 | 1115 | 126.1 |
| F6XTD9 | NUP155 | Nucleoporin 155 OS=Canis lupus familiaris OX=9615 GN=NUP155 PE=3 SV=1 | 4 | 4 | 4 | 4 | 1390 | 155 |
| E2RLQ9 | VCP | 15S Mg(2+)-ATPase p97 subunit OS=Canis lupus familiaris OX=9615 GN=VCP PE=3 SV=3 | 6 | 4 | 4 | 4 | 806 | 89.2 |
| A0A5F4DJ40 | PHB2 | Prohibitin OS=Canis lupus familiaris OX=9615 GN=PHB2 PE=3 SV=1 | 19 | 4 | 4 | 4 | 267 | 29.8 |
| E2RLS3 | HSP90AB1 | HATPase_c domain-containing protein OS=Canis lupus familiaris OX=9615 GN=HSP90AB1 PE=3 SV=2 | 9 | 6 | 6 | 3 | 724 | 83.2 |
| F6XV93 | LONP1 | Lon protease homolog, mitochondrial OS=Canis lupus familiaris OX=9615 GN=LONP1 PE=3 SV=2 | 4 | 3 | 3 | 3 | 960 | 106.6 |
| A0A5F4C634 | ATP2A2 | Sarcoplasmic/endoplasmic reticulum calcium ATPase 2 OS=Canis lupus familiaris OX=9615 GN=ATP2A2 PE=4 SV=1 | 4 | 3 | 3 | 3 | 1001 | 109.8 |
| F1PLG2 | NNT | Proton-translocating NAD(P)(+) transhydrogenase OS=Canis lupus familiaris OX=9615 GN=NNT PE=3 SV=3 | 4 | 3 | 3 | 3 | 1104 | 115.5 |
| A0A5F4C137 | SND1 | Staphylococcal nuclease domain-containing protein OS=Canis lupus familiaris OX=9615 GN=SND1 PE=4 SV=1 | 4 | 3 | 3 | 3 | 853 | 95.6 |

| | | | | | | | | |
|---|---|---|---|---|---|---|---|---|
| F1P8B4 | CANX | Calnexin OS=Canis lupus familiaris OX=9615 GN=CANX PE=3 SV=2 | 4 | 3 | 3 | 3 | 591 | 67.2 |
| F1PM56 | CPT1A | Carnitine O-palmitoyltransferase OS=Canis lupus familiaris OX=9615 GN=CPT1A PE=3 SV=1 | 4 | 3 | 3 | 3 | 773 | 88.1 |
| J9P425 | CD79A | 40S ribosomal protein S19 OS=Canis lupus familiaris OX=9615 GN=CD79A PE=3 SV=2 | 19 | 3 | 3 | 3 | 145 | 16.1 |
| F1Q0R0 | KRT14 | Keratin 14 OS=Canis lupus familiaris OX=9615 GN=KRT14 PE=3 SV=3 | 5 | 4 | 4 | 2 | 531 | 57.6 |
| J9NSW0 | TRAP1 | TNF receptor associated protein 1 OS=Canis lupus familiaris OX=9615 GN=TRAP1 PE=3 SV=2 | 5 | 3 | 3 | 2 | 832 | 93.3 |
| E2QYZ0 | ALDH18A1 | Delta-1-pyrroline-5-carboxylate synthase OS=Canis lupus familiaris OX=9615 GN=ALDH18A1 PE=3 SV=1 | 3 | 2 | 2 | 2 | 795 | 87.2 |
| A0A5F4CWM9 | MYH9 | Myosin-9 OS=Canis lupus familiaris OX=9615 GN=MYH9 PE=3 SV=1 | 1 | 2 | 2 | 2 | 1981 | 229 |
| F6Y258 | GTF2I | General transcription factor IIi OS=Canis lupus familiaris OX=9615 GN=GTF2I PE=4 SV=1 | 3 | 2 | 2 | 2 | 999 | 112.4 |
| A0A5F4BSL9 | | Uncharacterized protein OS=Canis lupus familiaris OX=9615 PE=3 SV=1 | 25 | 2 | 2 | 2 | 106 | 12.4 |
| E2RQ08 | RPN1 | Dolichyl-diphosphooligosaccharide--protein glycosyltransferase subunit 1 OS=Canis lupus familiaris OX=9615 GN=RPN1 PE=1 SV=1 | 4 | 2 | 2 | 2 | 607 | 68.5 |
| A0A5F4D104 | EGFR | Receptor protein-tyrosine kinase OS=Canis lupus familiaris OX=9615 GN=EGFR PE=4 SV=1 | 3 | 2 | 2 | 2 | 1138 | 126.9 |
| E2QW85 | | Elongation factor 1-alpha OS=Canis lupus familiaris OX=9615 PE=3 SV=2 | 5 | 2 | 2 | 2 | 461 | 49.9 |

| | | | | | | | | |
|---|---|---|---|---|---|---|---|---|
| F2Z4Q6 | AFP | Alpha fetoprotein OS=Canis lupus familiaris OX=9615 GN=AFP PE=4 SV=2 | 2 | 2 | 2 | 2 | 637 | 71.9 |
| F6XRY2 | EEF2 | Eukaryotic translation elongation factor 2 OS=Canis lupus familiaris OX=9615 GN=EEF2 PE=3 SV=1 | 3 | 2 | 2 | 2 | 858 | 95.3 |
| L7N0I7 | TUBB4B | Tubulin beta chain OS=Canis lupus familiaris OX=9615 GN=TUBB4B PE=3 SV=1 | 5 | 2 | 2 | 2 | 445 | 49.8 |
| Q6TEQ7 | ANXA2 | Annexin A2 OS=Canis lupus familiaris OX=9615 GN=ANXA2 PE=1 SV=1 | 6 | 2 | 2 | 2 | 339 | 38.6 |
| A0A5F4DI88 | PRDX4 | Peroxiredoxin 4 OS=Canis lupus familiaris OX=9615 GN=PRDX4 PE=4 SV=1 | 8 | 2 | 2 | 2 | 257 | 29.3 |
| A0A5F4BSS2 | HSPA9 | 75 kDa glucose-regulated protein OS=Canis lupus familiaris OX=9615 GN=HSPA9 PE=3 SV=1 | 4 | 2 | 2 | 2 | 625 | 67.4 |
| Q28298 | RRBP1 | Ribosome-binding protein 1 OS=Canis lupus familiaris OX=9615 GN=RRBP1 PE=2 SV=1 | 1 | 2 | 2 | 2 | 1534 | 164.5 |
| F1PPF7 | HK1 | Hexokinase OS=Canis lupus familiaris OX=9615 GN=HK1 PE=3 SV=3 | 2 | 2 | 2 | 2 | 935 | 104.2 |
| E2RI34 | RPL27A | 60S ribosomal protein L27a OS=Canis lupus familiaris OX=9615 GN=RPL27A PE=3 SV=1 | 14 | 2 | 2 | 2 | 148 | 16.6 |
| A0A5F4D3H8 | DDOST | Dolichyl-diphosphooligosaccharide--protein glycosyltransferase 48 kDa subunit OS=Canis lupus familiaris OX=9615 GN=DDOST PE=3 SV=1 | 5 | 2 | 2 | 2 | 442 | 49.1 |
| F1Q0N9 | KRT14 | Keratin 14 OS=Canis lupus familiaris OX=9615 GN=KRT14 PE=3 SV=2 | 5 | 3 | 3 | 1 | 494 | 52.9 |
| F1PGY1 | HSP90AA1 | Heat shock protein 90 alpha family class A member 1 OS=Canis lupus familiaris OX=9615 GN=HSP90AA1 PE=3 SV=3 | 5 | 2 | 2 | 1 | 542 | 63.6 |

| | | | | | | | | |
|---|---|---|---|---|---|---|---|---|
| E2RNT3 | DHX9 | DEAH box protein 9 OS=Canis lupus familiaris OX=9615 GN=DHX9 PE=3 SV=1 | 1 | 1 | 2 | 1 | 1276 | 141.2 |
| O18740 | KRT9 | Keratin, type I cytoskeletal 9 OS=Canis lupus familiaris OX=9615 GN=KRT9 PE=3 SV=1 | 2 | 2 | 2 | 1 | 786 | 76.3 |
| A0A5F4BRW6 | POR | NADPH--hemoprotein reductase OS=Canis lupus familiaris OX=9615 GN=POR PE=4 SV=1 | 1 | 1 | 2 | 1 | 656 | 74.2 |
| A0A5F4CUN5 | KTN1 | Kinectin 1 OS=Canis lupus familiaris OX=9615 GN=KTN1 PE=4 SV=1 | 1 | 1 | 1 | 1 | 1401 | 160.3 |
| F1P6B7 | ANXA1 | Annexin OS=Canis lupus familiaris OX=9615 GN=ANXA1 PE=3 SV=1 | 3 | 1 | 1 | 1 | 345 | 38.6 |
| J9P621 | ATP5O | ATP synthase peripheral stalk subunit OSCP OS=Canis lupus familiaris OX=9615 GN=ATP5O PE=2 SV=1 | 7 | 1 | 1 | 1 | 213 | 23.5 |
| J9P9Z7 | RPS20 | 40S ribosomal protein S20 OS=Canis lupus familiaris OX=9615 GN=RPS20 PE=3 SV=1 | 9 | 1 | 1 | 1 | 119 | 13.4 |
| F1Q1M2 | IARS2 | Isoleucyl-tRNA synthetase OS=Canis lupus familiaris OX=9615 GN=IARS2 PE=3 SV=3 | 1 | 1 | 1 | 1 | 963 | 108.1 |
| F1PLR0 | RACK1 | Receptor for activated C kinase 1 OS=Canis lupus familiaris OX=9615 GN=RACK1 PE=4 SV=2 | 4 | 1 | 1 | 1 | 317 | 35.1 |
| A0A5F4CGI3 | DDX5 | DEAD box protein 5 OS=Canis lupus familiaris OX=9615 GN=DDX5 PE=3 SV=1 | 2 | 1 | 1 | 1 | 535 | 60.5 |
| E2REU6 | KRT18 | IF rod domain-containing protein OS=Canis lupus familiaris OX=9615 GN=KRT18 PE=3 SV=3 | 3 | 1 | 1 | 1 | 431 | 48.2 |
| F1Q342 | LOC489992 | SpaA domain-containing protein OS=Canis lupus familiaris OX=9615 GN=LOC489992 PE=4 SV=2 | 1 | 1 | 1 | 1 | 1132 | 124.5 |

| | | | | | | | | |
|---|---|---|---|---|---|---|---|---|
| F2Z4P9 | RAB10 | Ras-related protein Rab-10 OS=Canis lupus familiaris OX=9615 GN=RAB10 PE=4 SV=1 | 6 | 1 | 1 | 1 | 200 | 22.5 |
| F1Q3V2 | ACLY | ATP-citrate synthase OS=Canis lupus familiaris OX=9615 GN=ACLY PE=3 SV=3 | 1 | 1 | 1 | 1 | 1134 | 123.9 |
| J9NXR3 | ITGB4 | Integrin beta OS=Canis lupus familiaris OX=9615 GN=ITGB4 PE=3 SV=2 | 1 | 1 | 1 | 1 | 1766 | 196.2 |
| F6V9R9 | GLG1 | Golgi apparatus protein 1 OS=Canis lupus familiaris OX=9615 GN=GLG1 PE=4 SV=1 | 1 | 1 | 1 | 1 | 1186 | 134.9 |
| P81709 | | Lysozyme C, spleen isozyme OS=Canis lupus familiaris OX=9615 PE=1 SV=1 | 9 | 1 | 1 | 1 | 130 | 14.6 |
| F1PEN6 | TFRC | Transferrin receptor protein 1 OS=Canis lupus familiaris OX=9615 GN=TFRC PE=3 SV=2 | 1 | 1 | 1 | 1 | 918 | 100.9 |
| A0A5F4C4D4 | LETM1 | Leucine zipper-EF-hand-containing transmembrane protein 1 OS=Canis lupus familiaris OX=9615 GN=LETM1 PE=3 SV=1 | 2 | 1 | 1 | 1 | 765 | 85.7 |
| E2R580 | VAPA | VAMP associated protein A OS=Canis lupus familiaris OX=9615 GN=VAPA PE=4 SV=1 | 5 | 1 | 1 | 1 | 249 | 27.8 |
| A0A5F4CNG7 | DSP | Desmoplakin OS=Canis lupus familiaris OX=9615 GN=DSP PE=4 SV=1 | 0 | 1 | 1 | 1 | 2057 | 236.2 |
| A0A5F4CJR2 | HYOU1 | Hypoxia up-regulated 1 OS=Canis lupus familiaris OX=9615 GN=HYOU1 PE=3 SV=1 | 2 | 1 | 1 | 1 | 678 | 75.3 |
| E2R4F5 | RPL13 | 60S ribosomal protein L13 OS=Canis lupus familiaris OX=9615 GN=RPL13 PE=3 SV=1 | 5 | 1 | 1 | 1 | 211 | 24.3 |
| A0A5F4C6J8 | RPL22 | Uncharacterized protein OS=Canis lupus familiaris OX=9615 GN=RPL22 PE=3 SV=1 | 10 | 1 | 1 | 1 | 128 | 14.8 |

| | | | | | | | | |
|---|---|---|---|---|---|---|---|---|
| A0A5F4BTP4 | AP1B1 | AP complex subunit beta OS=Canis lupus familiaris OX=9615 GN=AP1B1 PE=3 SV=1 | 1 | 1 | 1 | 1 | 938 | 103.5 |
| E2QWK2 | PNPT1 | Polynucleotide phosphorylase 1 OS=Canis lupus familiaris OX=9615 GN=PNPT1 PE=3 SV=3 | 1 | 1 | 1 | 1 | 763 | 83.7 |
| A0A5F4CT43 | PDHA1 | Pyruvate dehydrogenase E1 component subunit alpha OS=Canis lupus familiaris OX=9615 GN=PDHA1 PE=4 SV=1 | 3 | 1 | 1 | 1 | 405 | 44.9 |
| F1Q3W0 | HNRNPU | Heterogeneous nuclear ribonucleoprotein U OS=Canis lupus familiaris OX=9615 GN=HNRNPU PE=4 SV=3 | 1 | 1 | 1 | 1 | 805 | 88.3 |
| F1PTZ5 | CD44 | CD44 antigen OS=Canis lupus familiaris OX=9615 GN=CD44 PE=4 SV=3 | 1 | 1 | 1 | 1 | 783 | 85.7 |
| A0A5F4BYR1 | UGGT1 | UDP-glucose glycoprotein glucosyltransferase 1 OS=Canis lupus familiaris OX=9615 GN=UGGT1 PE=3 SV=1 | 1 | 1 | 1 | 1 | 1557 | 177.9 |
| A0A5F4BU46 | H1-2 | Histone H2B OS=Canis lupus familiaris OX=9615 GN=H1-2 PE=3 SV=1 | 3 | 1 | 1 | 1 | 435 | 45.7 |
| Q9XSU7 | RPL27 | 60S ribosomal protein L27 OS=Canis lupus familiaris OX=9615 GN=RPL27 PE=2 SV=3 | 7 | 1 | 1 | 1 | 136 | 15.8 |
| A0A5F4CJY9 | AFG3L2 | AFG3 like matrix AAA peptidase subunit 2 OS=Canis lupus familiaris OX=9615 GN=AFG3L2 PE=3 SV=1 | 1 | 1 | 1 | 1 | 732 | 81.2 |
| E2RIA8 | RPL8 | 60S ribosomal protein L8 OS=Canis lupus familiaris OX=9615 GN=RPL8 PE=3 SV=1 | 4 | 1 | 1 | 1 | 257 | 28 |
| A0A5F4C2R0 | CTNND1 | Uncharacterized protein OS=Canis lupus familiaris OX=9615 GN=CTNND1 PE=3 SV=1 | 1 | 1 | 1 | 1 | 896 | 100.2 |
| E2R667 | COPB2 | Coatomer subunit beta' OS=Canis lupus familiaris OX=9615 GN=COPB2 PE=3 SV=1 | 1 | 1 | 1 | 1 | 906 | 102.3 |

| | | | | | | | | |
|---|---|---|---|---|---|---|---|---|
| F1PCE8 | LOC475521 | Peptidase S1 domain-containing protein OS=Canis lupus familiaris OX=9615 GN=LOC475521 PE=4 SV=1 | 4 | 1 | 1 | 1 | 246 | 26.3 |
| E2RS49 | RPS25 | 40S ribosomal protein S25 OS=Canis lupus familiaris OX=9615 GN=RPS25 PE=3 SV=3 | 8 | 1 | 1 | 1 | 125 | 13.7 |
| E2RB37 | PDIA6 | Protein disulfide-isomerase A6 OS=Canis lupus familiaris OX=9615 GN=PDIA6 PE=3 SV=1 | 3 | 1 | 1 | 1 | 440 | 48.3 |
| A0A5F4CXM3 | TAOK3 | TAO kinase 3 OS=Canis lupus familiaris OX=9615 GN=TAOK3 PE=4 SV=1 | 1 | 1 | 1 | 1 | 840 | 98.6 |
| J9P4G7 | C17H1orf68 | Chromosome 17 C1orf68 homolog OS=Canis lupus familiaris OX=9615 GN=C17H1orf68 PE=4 SV=2 | 3 | 1 | 1 | 1 | 271 | 29.1 |
| A0A5F4C5B7 | SEC24C | SEC24 homolog C, COPII coat complex component OS=Canis lupus familiaris OX=9615 GN=SEC24C PE=3 SV=1 | 1 | 1 | 1 | 1 | 1093 | 118.6 |
| F1PAA9 | CDH1 | Cadherin-1 OS=Canis lupus familiaris OX=9615 GN=CDH1 PE=1 SV=2 | 1 | 1 | 1 | 1 | 885 | 97.7 |
| A0A140T8E0 | CAPRIN1 | Cell cycle associated protein 1 OS=Canis lupus familiaris OX=9615 GN=CAPRIN1 PE=3 SV=1 | 2 | 1 | 1 | 1 | 717 | 78.8 |
| E2RFR0 | RPS8 | 40S ribosomal protein S8 OS=Canis lupus familiaris OX=9615 GN=RPS8 PE=3 SV=1 | 6 | 1 | 1 | 1 | 208 | 24.2 |
| E2R4L7 | SHMT2 | Serine hydroxymethyltransferase OS=Canis lupus familiaris OX=9615 GN=SHMT2 PE=3 SV=3 | 2 | 1 | 1 | 1 | 549 | 61.3 |
| F2Z4P2 | RPL7A | 60S ribosomal protein L7a OS=Canis lupus familiaris OX=9615 GN=RPL7A PE=3 SV=3 | 4 | 1 | 1 | 1 | 266 | 30 |
| E2RQP6 | EIF3C | Eukaryotic translation initiation factor 3 subunit C OS=Canis lupus familiaris OX=9615 GN=EIF3C PE=3 SV=2 | 1 | 1 | 1 | 1 | 942 | 108.9 |

| | | | | | | | | |
|---|---|---|---|---|---|---|---|---|
| J9PA20 | GLUD1 | Glutamate dehydrogenase (NAD(P)(+)) OS=Canis lupus familiaris OX=9615 GN=GLUD1 PE=3 SV=2 | 2 | 1 | 1 | 1 | 572 | 62.2 |
| A0A5F4CD08 | SEC11A | Signal peptidase complex catalytic subunit SEC11 OS=Canis lupus familiaris OX=9615 GN=SEC11A PE=3 SV=1 | 4 | 1 | 1 | 1 | 183 | 21 |
| F1PK90 | DDX46 | RNA helicase OS=Canis lupus familiaris OX=9615 GN=DDX46 PE=4 SV=2 | 1 | 1 | 1 | 1 | 1032 | 117.4 |
| A0A5F4C0Z8 | GOLGA2 | GOLGA2L5 domain-containing protein OS=Canis lupus familiaris OX=9615 GN=GOLGA2 PE=4 SV=1 | 1 | 1 | 1 | 1 | 987 | 111.5 |
| E2R0B6 | PSMB6 | Proteasome subunit beta OS=Canis lupus familiaris OX=9615 GN=PSMB6 PE=3 SV=1 | 5 | 1 | 1 | 1 | 239 | 25.5 |
| E2RNY7 | ATP5F1C | ATP synthase subunit gamma OS=Canis lupus familiaris OX=9615 GN=ATP5F1C PE=2 SV=3 | 4 | 1 | 1 | 1 | 298 | 33 |
| F1Q4J0 | GANAB | Glucosidase II alpha subunit OS=Canis lupus familiaris OX=9615 GN=GANAB PE=3 SV=2 | 1 | 1 | 1 | 1 | 944 | 106.3 |
| F1PDG4 | UBB | Uncharacterized protein OS=Canis lupus familiaris OX=9615 GN=UBB PE=4 SV=2 | 21 | 1 | 1 | 1 | 229 | 25.7 |
| E2RR58 | RPL12 | 60S ribosomal protein L12 OS=Canis lupus familiaris OX=9615 GN=RPL12 PE=1 SV=1 | 5 | 1 | 1 | 1 | 165 | 17.8 |
| E2R8R8 | TFPT | 40S ribosomal protein S9 OS=Canis lupus familiaris OX=9615 GN=TFPT PE=3 SV=2 | 3 | 1 | 1 | 1 | 228 | 26.3 |
| F1PIP0 | HADHA | Enoyl-CoA hydratase OS=Canis lupus familiaris OX=9615 GN=HADHA PE=3 SV=3 | 2 | 1 | 1 | 1 | 762 | 82.9 |
| A0A5F4BWP4 | SPATA7 | Spermatogenesis associated 7 OS=Canis lupus familiaris OX=9615 GN=SPATA7 PE=4 SV=1 | 1 | 1 | 1 | 1 | 563 | 64.1 |

| | | | | | | | | | |
|---|---|---|---|---|---|---|---|---|---|
| E2R4X3 | DAD1 | Dolichyl-diphosphooligosaccharide--protein glycosyltransferase subunit DAD1 OS=Canis lupus familiaris OX=9615 GN=DAD1 PE=1 SV=2 | 10 | 1 | 1 | 1 | 119 | 13.1 |
| E2RHG2 | PRDX1 | Peroxiredoxin-1 OS=Canis lupus familiaris OX=9615 GN=PRDX1 PE=3 SV=2 | 5 | 1 | 1 | 1 | 220 | 24.3 |
| E2QYG6 | ATP6V1A | H(+)-transporting two-sector ATPase OS=Canis lupus familiaris OX=9615 GN=ATP6V1A PE=3 SV=1 | 1 | 1 | 1 | 1 | 618 | 68.4 |
| F1P6U7 | LRRC40 | Leucine rich repeat containing 40 OS=Canis lupus familiaris OX=9615 GN=LRRC40 PE=4 SV=3 | 5 | 1 | 1 | 1 | 552 | 62.9 |

**Table S2.** Primers for sgRNA for gene knockdown.

| **Primer** | **Sequences (5'-3')** |
|---|---|
| sgRNA | GTTTGCACTAATCGTCTACT-GGG |
| PD-L1 F | AGAGATGAGATCCAGGGTTCCA |
| PD-L1 R | TAGCCGATCAAGCAGCAGTAAA |

PCR program: initial denaturation for 3 min at 94°C, 35 cycles at 94 °C for 30s, 62 °C for 35s, 72 °C for 35s, and a final extension step of 5 min at 72°C.
